# Exposure of *Candida albicans* β (1,3)-glucan is promoted by activation of the Cek1 pathway

**DOI:** 10.1371/journal.pgen.1007892

**Published:** 2019-01-31

**Authors:** Tian Chen, Joseph W. Jackson, Robert N. Tams, Sarah E. Davis, Timothy E. Sparer, Todd B. Reynolds

**Affiliations:** Department of Microbiology, University of Tennessee, Knoxville, TN, United States of America; Max-Planck-Institut fur Evolutionsbiologie, GERMANY

## Abstract

*Candida albicans* is among the most common causes of human fungal infections and is an important source of mortality. *C*. *albicans* is able to diminish its detection by innate immune cells through masking of β (1,3)-glucan in the inner cell wall with an outer layer of heavily glycosylated mannoproteins (mannan). However, mutations or drugs that disrupt the cell wall can lead to exposure of β (1,3)-glucan (unmasking) and enhanced detection by innate immune cells through receptors like Dectin-1, the C-type signaling lectin. Previously, our lab showed that the pathway for synthesizing the phospholipid phosphatidylserine (PS) plays a role in β (1,3)-glucan masking. The homozygous PS synthase knockout mutant, *cho1Δ/Δ*, exhibits increased exposure of β (1,3)-glucan. Several Mitogen Activated Protein Kinase (MAPK) pathways and their upstream Rho-type small GTPases are important for regulating cell wall biogenesis and remodeling. In the *cho1Δ/Δ* mutant, both the Cek1 and Mkc1 MAPKs are constitutively activated, and they act downstream of the small GTPases Cdc42 and Rho1, respectively. In addition, Cdc42 activity is up-regulated in *cho1Δ/Δ*. Thus, it was hypothesized that activation of Cdc42 or Rho1 and their downstream kinases cause unmasking. Disruption of *MKC1* does not decrease unmasking in *cho1Δ/Δ*, and hyperactivation of Rho1 in wild-type cells increases unmasking and activation of both Cek1 and Mkc1. Moreover, independent hyperactivation of the MAP kinase kinase kinase Ste11 in wild-type cells leads to Cek1 activation and increased β (1,3)-glucan exposure. Thus, upregulation of the Cek1 MAPK pathway causes unmasking, and may be responsible for unmasking in *cho1Δ/Δ*.

## Author summary

*Candida albicans* causes fungal infections in the oral cavities and bloodstreams of patients with weakened immune function, such as AIDS or cancer patients. The immune system detects fungal infections, in part, by detecting the antigenic cell wall polysaccharide β (1,3)-glucan. The ability to mask β (1,3)-glucan from immune detection is a virulence factor of *C*. *albicans* and a range of fungal pathogens. If synthesis of the phospholipid phosphatidylserine is disrupted in *C*. *albicans* (*cho1Δ/Δ* mutation), then *cho1Δ/Δ* exhibits significantly increased exposure of β (1,3)-glucan to immune detection compared to wild-type. Intracellular signaling cascades that regulate cell wall synthesis are upregulated in the *cho1Δ/Δ* mutant. It was hypothesized that upregulation of these pathways might be responsible for unmasking in this mutant. Genetic approaches were used to activate these pathways independently of the *cho1Δ/Δ* mutation. It was discovered that activation of one pathway, Cdc42-Cek1, leads to β (1,3)-glucan exposure. Thus, this pathway can cause β(1,3)-glucan exposure, and its upregulation may be the cause of unmasking in the *cho1Δ/Δ* mutant.

## Introduction

*Candida albicans* is a human commensal that is part of the natural flora of the oral, genital and gastrointestinal tracts. *Candida* species are also the most common fungal pathogens of humans and cause diseases ranging from superficial infections of mucosal surfaces to severe systemic bloodstream infections in immune-compromised patients [1–4], with a mortality rate of approximately 30% [2]. Three major classes of antifungals are used to treat systemic infections including azoles, echinocandins, and polyenes [5–7]. However, drug resistance or toxicity has put limits on these agents.

The *C*. *albicans* cell wall is considered a good therapeutic drug target due to its role in fungal pathogenicity as it presents important virulence factors, antigenic cell wall proteins and polysaccharides, and serves as the intermediate for fungal-host interactions [3, 8, 9]. One potential method for improving anti-fungal strategies could be to enhance the detection of fungal cell wall antigens by host immune cells. A major innate immune receptor for fungi like *C*. *albicans* is Dectin-1, a C-type signaling lectin that can recognize β (1,3)-glucan, which is an important component of fungal cell walls [8, 10, 11]. This recognition can initiate protective antifungal immune responses in innate immune cells like macrophages, dendritic cells and neutrophils. The fungal cell wall consists of an inner layer that is enriched in β (1,3)-glucan and underlying chitin, and an outer layer of mannosylated proteins [8]. Under normal conditions, *C*. *albicans* masks β (1,3)-glucan from Dectin-1 detection via the outer layer of mannosylated proteins [12, 13]. However, unmasking of β (1,3)-glucan can be induced through treatments with drugs such as echinocandins [12] or by certain genetic mutations that disrupt cell wall integrity[12–15].

It has been previously reported that the phosphatidylserine (PS) synthase enzyme (Cho1) controls cell wall β (1,3)-glucan exposure [13]. Phospholipids are crucial components of cellular membranes in eukaryotes. Cho1 synthesizes PS that can act as an end product, but also can be further decarboxylated to form phosphatidylethanolamine (PE). PS and PE are both essential for *C*. *albicans* virulence [16]. We found that the homozygous *CHO1* mutant, *cho1Δ/Δ*, exhibits greater β (1,3)-glucan exposure compared to wild-type [13, 14]. This exposure allows increased recognition by Dectin-1 and elicits a stronger pro-inflammatory response [13, 14, 17]. However, the detailed mechanism by which β (1,3)-glucan exposure is caused by *CHO1* disruption remains unknown.

**Fig 1:**
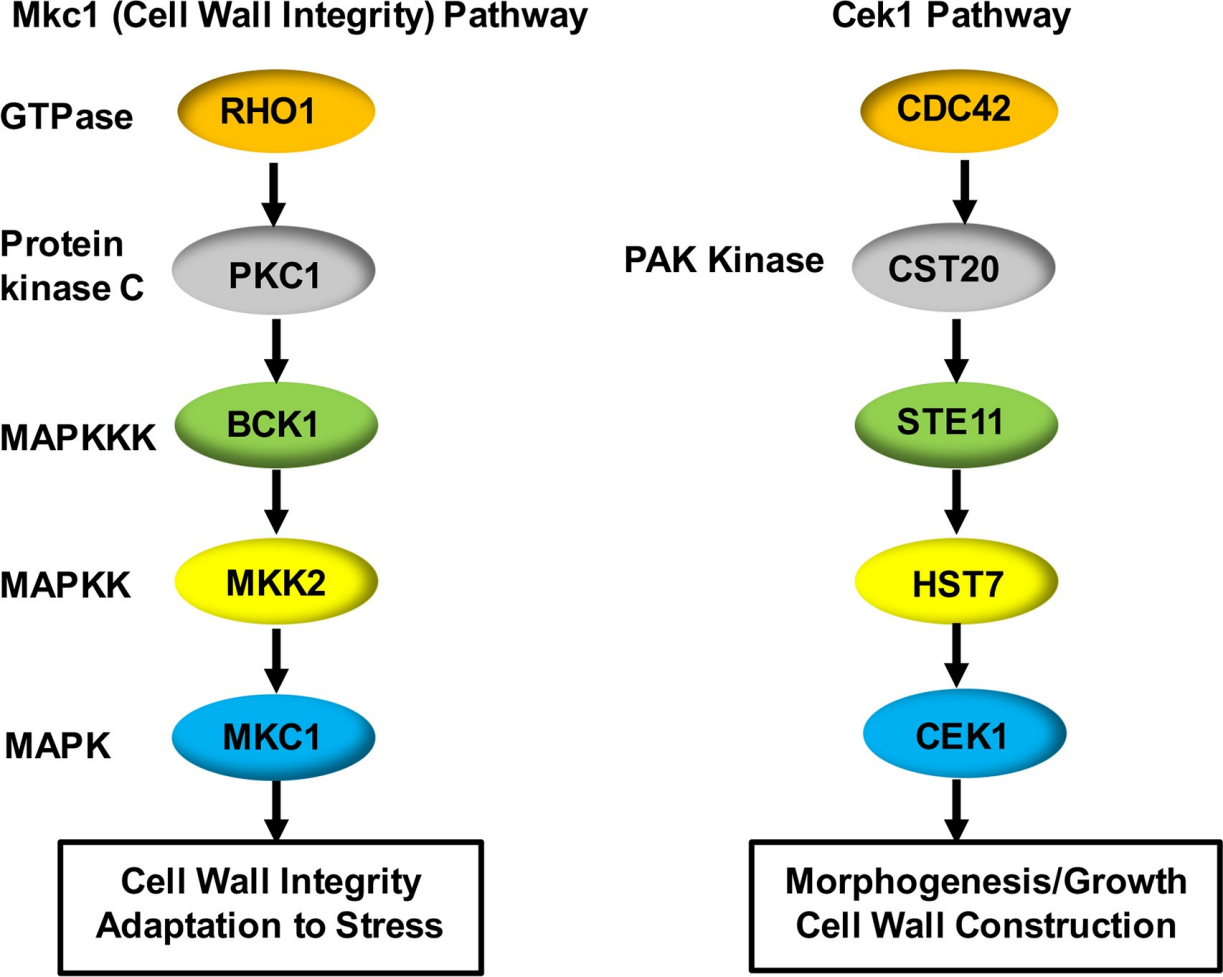
The Cek1 and Mkc1 MAP kinase signaling cascades in *C*. *albicans* are involved in cell wall biogenesis. The Cek1 and Mkc1 MAP kinase cascades, and their respective upstream activator signaling proteins are shown. Rho1 activates protein kinase C (Pkc1), which activates the Mkc1 MAP kinase cascade. Cdc42 activates the PAK kinase Cst20 which activates the Cek1 MAP kinase cascade.

The process of cell wall biogenesis and remodeling is governed through complex signaling pathways, including several mitogen-activated protein kinase (MAPK) cascades and their upstream Rho-type GTPases (Fig 1). MAPK pathways are conserved signaling cascades in eukaryotes that are important for dealing with a wide range of stimuli, including osmotic stress, oxidative stress, cell wall damage, and changes in glycosylation [9, 15, 17–19]. This signaling cascade is composed of a conserved module of three kinases: the MAP kinase kinase kinase (MAPKKK), the MAP kinase kinase (MAPKK) and the MAP kinase (MAPK). The MAPK activates downstream transcription factors and effectors to initiate gene expression for better adaptation to the environment [19]. Among these MAPK pathways, Ste11-Hst7-Cek1 composes the Cek1 MAPK cascade, and is reported to control β (1,3)-glucan masking in *C*. *albicans* [15, 20, 21]. *CEK1* null mutants display unmasking of β (1,3)-glucan and hyper-sensitivity to agents that disturb the cell wall such as Congo red [15]. The Mkc1 MAPK route, consisting of Bck1-Mkk2-Mkc1, is primarily involved in cell wall construction, as well as responding to exogenous cell wall stress, oxidative stimuli, antifungal drugs, and low-temperature shocks [22, 23]. Yet, this pathway does not appear to be required for masking in *C*. *albicans* [24], although it is hypersensitive to specific cell wall insults such as echinocandins or calcofluor white.

The upstream small GTPases Cdc42 and Rho1 transmit the signal toward Cek1- and Mkc1- associated MAPK cascades, respectively (Fig 1) [18, 23]. They are also important in remodeling the rigid structure of the cell wall during vegetative growth and morphogenesis [25]. Rho1 is a well-known major regulator of the cell wall integrity signaling cascade through several downstream effectors [23, 25–30]. Rho1 is also the regulatory subunit of β (1,3)-glucan synthase, and therefore directly controls cell wall biosynthesis via the binding and activation of its catalytic subunits, such as Fks1 [26, 31]. Cdc42 is essential for cellular polarized growth, and it acts on a variety of downstream effector proteins in *C*. *albicans*, including kinases such as PAK kinase family members Cst20/Cla4 [32–37].

Given the role of the GTPase-associated signaling pathways in cell wall remodeling and regulation, we studied the impact of these signaling routes in affecting β (1,3)-glucan masking in the *C*. *albicans cho1Δ/Δ* PS synthase mutant. We found that in the *cho1Δ/Δ* mutant there is upregulation of the activity of both Cek1 and Mkc1 MAPKs. Furthermore, we present data indicating that activation of the Cek1 pathway, in particular, is sufficient to cause β(1,3)-glucan exposure in the *cho1Δ/Δ* mutant.

## Results

### *C*. *albicans cho1Δ/Δ* exhibits activated MAPKs

**Fig 2:**
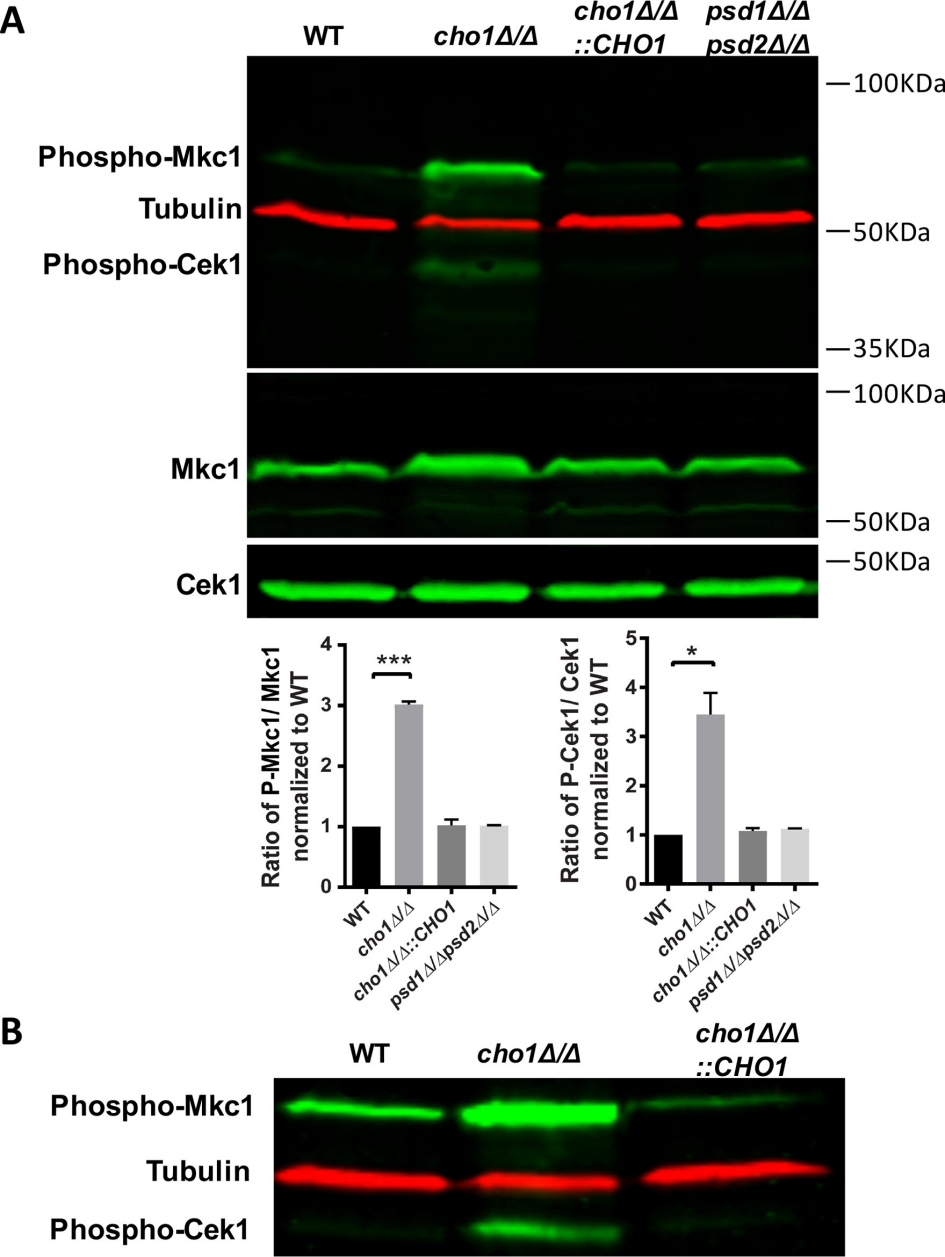
Cek1 and Mkc1 MAPKs exhibit increased activation in *cho1Δ/Δ* cells compared to wild-type. (A) Proteins from yeast-form cells growing in log phase in YPD media were extracted and Western blotting was performed with anti-phospho-p44/42 antibody to detect Phospho-Mkc1 and Phospho-Cek1. Anti-Mkc1 was used for total Mkc1, anti-Cek1 for total Cek1, and anti-tubulin as a loading control. Graphs of quantification by Image J of the phosphorylated forms of each kinase are expressed as a percent of the wild-type control after being normalized to the total kinase blot for each respective MAP kinase and the tubulin loading controls for each gel. Quantification is based on three biological replicates. The statistical analysis was performed by using One-way ANOVA *, p = 0.0308. ***, p = 0.0005. (B) Western blotting was performed on extracts from cells grown as hyphae in RPMI media and probed with antibodies detecting Phospho-Cek1 and Phospho-Mkc1 as well as the tubulin loading control.

Given the strong cell wall phenotypes seen in *cho1Δ/Δ*, we hypothesized that this mutant might exhibit increased activation of cell wall signaling pathways such as Cek1 and Mkc1 MAP kinase cascades. As shown in Fig 2, Western blots with the Phospho-p42/44 antibody, that labels the phosphorylated (activated) forms of both Cek1 and Mkc1, revealed that these kinases were constitutively phosphorylated in *cho1Δ/Δ* compared to wild-type and other test strains. No significant difference was found between the *psd1Δ/Δpsd2Δ/Δ* mutant (synthesizes PE from PS) and wild-type (Fig 2A). This indicates that disruption of the PS synthase specifically up-regulates the activity of both cell wall MAPK cascades. Similar trends were also seen under hyphal induction conditions. When cells were sub-cultured in RPMI 1640 medium (induces filamentation [38]), *cho1Δ/Δ* exhibited greater phosphorylation of Cek1 and Mkc1 than wild-type and other test strains (Fig 2B). Collectively, these results indicate that loss of Cho1 activates the Cek1 and Mkc1 MAPK pathways.

### Activation of the Cek1 pathway causes β (1,3)-glucan exposure

**Fig 3:**
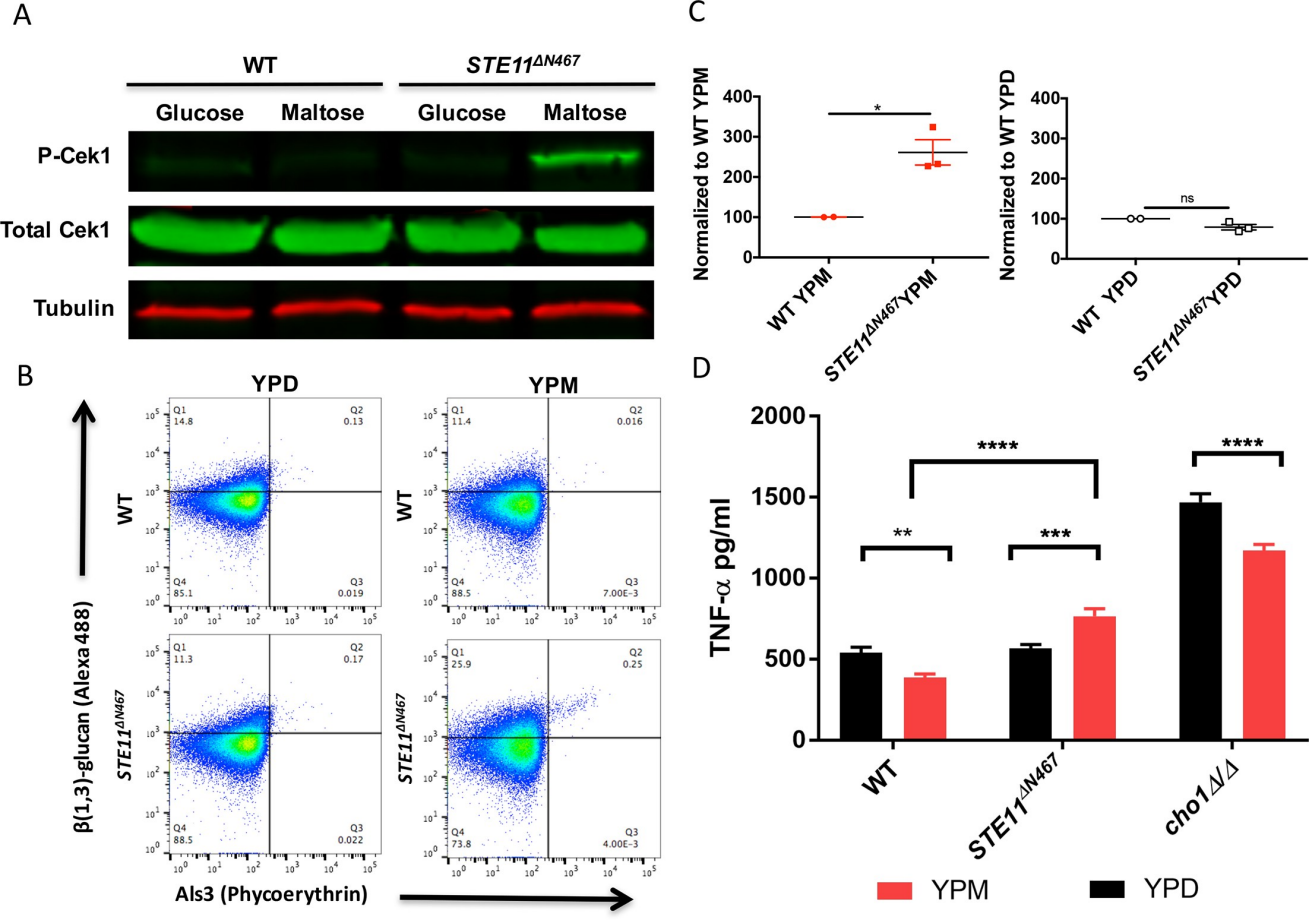
Hyperactive Ste11 (*STE11*^*ΔN467*^) causes significant increases in β (1,3)-glucan exposure and TNF-α secretion. (A) The Cek1 MAPK is hyper-activated by transforming wild-type cells with a hyperactive allele of *STE11* (*P*_*MAL*_*-STE11*^*ΔN467*^), which is induced by adding maltose as a carbon source. (B) Both wild-type and *STE11*^*ΔN467*^ expressing cells were cultured overnight (16hrs) in YPD or YPM individually and then were doubly stained. The Y-axis represents staining with soluble Dectin-1-Fc (sDectin-1-Fc) that binds to exposed β (1,3)-glucan, and the X-axis represents anti-Als3 antibody, which binds to the hyphal-specific protein Als3. Secondary antibodies were used for fluorescence as described in the Methods. Flow cytometry was performed to quantify β (1,3)-glucan exposure from the yeast-form population (Q1: sDectin-1 single positive staining; Q2: sDectin-1 & Als3 double positive staining; Q3: Als3 single positive staining; Q4: double negative staining). Gates were established with an unstained control where 97% of unstained cells gated within Q4. Gating strategies are further described in the Methods. (C) Left graph: Comparison of β (1,3)-glucan exposure from the yeast-form population of *STE11*^*ΔN467*^ versus that of wild-type, both of which are cultured in YPM. Data were compared by unpaired t-test *, p = 0.0289. Right graph: β (1,3)-glucan exposure was compared between these two strains when grown in YPD medium. (D) Expression of *STE11*^*ΔN467*^ significantly induces TNF-α secretion after growing in YPM overnight. RAW264.7 macrophages were challenged with various *C*. *albicans* stains. *C*. *albicans* strains were grown in YPD or YPM, washed, UV-inactivated, and then add to the macrophages for 4hrs. The macrophage supernatant was collected and assayed by ELISA to quantify TNF-α production. **, P = 0.0030; ***, P = 0.0002; ****, p<0.0001.

Galán-Díez *et al*. observed that a *cek1Δ/Δ* homozygous deletion mutant exhibits β (1,3)-glucan exposure in *C*. *albicans* [15]. In contrast, Li *et al*. reported that Cek1-inducing conditions, such as incubation with N-acetylglucosamine (GlcNAc) in the media, causes increased β (1,3)-glucan exposure in *C*. *albicans* [39]. To further investigate if activation of the Cek1 pathway increases exposure of β (1,3)-glucan in *C*. *albicans* yeast-form cells, we constructed a strain that expresses a hyper-active allele of *STE11* (*STE11^ΔN467^*) under the regulation of the maltose promoter (*P*_*MAL2*_). Deletion of 467 N-terminal amino acids, including the inhibitory domain of Ste11, hyper-activates this kinase [40]. Ste11 is upstream of Cek1, and activates it via sequential phosphorylation through Hst7 (Fig 1). Expression of the *STE11Δ*^*N467*^ allele in YP maltose (YPM) media results in greater phosphorylation of Cek1 compared to growth of this strain in YPD (represses *STE11*^*ΔN467*^ expression) (Fig 3A). The *STE11*^*ΔN467*^ expressing strain exhibited greater β (1,3)-glucan exposure in YPM than YPD when stained with anti- β (1,3)-glucan antibody (S1 Fig).

The Cek1 pathway is involved in inducing the yeast-to-hyphae transition. A small subset of cells form filaments in the hyper-activated *STE11^ΔN467^* strain in YPM, and hyphae exhibit β (1,3)-glucan unmasking more readily than yeast-form cells [12, 13]. To determine if the yeast-form cells themselves exhibited greater unmasking, we used flow cytometry with a second hyphal-specific probe to gate out hyphal cells while measuring β (1,3)-glucan exposure. In particular, we stained strains with soluble Dectin-1 (sDectin-1) protein which binds with exposed β (1,3)-glucan and anti-Als3 antibody, which stains the hyphal-specific protein Als3. Thus, Als3 staining was used as a marker to gate out hyphae by flow cytometry allowing us to focus on yeast-form cells. This double staining revealed that wild-type yeast-form cells expressing hyper-activated Ste11 (*STE11Δ*^*N467*^) in YPM have significantly increased unmasking compared to yeast cells in YPD (Fig 3B); compare the 1^st^ quadrants (Q1) of the plots of *STE11^ΔN467^* grown in both YPM and YPD (bottom two plots).

β-1,3-glucan exposure is more intense at bud scars, which presented the possibility that the higher glucan exposure in *STE11^ΔN467^*-YPM is associated with more bud scars provided that maltose increases growth rate. In fact, a growth curve demonstrated that both wild-type and *STE11^ΔN467^* cells cultured in YPM grew slightly better compared to corresponding strains in YPD culture (S2 Fig). However, when we co-stained cells with β (1,3)-glucan antibody and calcofluor white, a dye that stains the chitin that is normally concentrated at the bud scar [41], the exposed β (1,3)-glucan in *STE11^ΔN467^* is scattered along the cell periphery, whereas the calcofluor white staining is constricted to the division sites (*e*. *g*. bud scars), revealing little overlap (S3 Fig). Furthermore, *STE11^ΔN467^* and wild-type have similar growth rates in YPM (S2 Fig), but *STE11^ΔN467^* has significantly elevated β (1,3)-glucan unmasking in this medium (Fig 3B and 3C). Conversely, the strains have similar rates of growth and β(1,3)-glucan exposure in YPD (Fig 3B & 3C), a condition where Cek1 is not hyperactived (Fig 3A). Moreover, wild-type replicates more rapidly in YPM than YPD, but β(1,3)-glucan exposure is comparable for wild-type in both media (Fig 3B). Altogether, these data indicate that hyperactivation of the Cek1 pathway leads to increased β (1,3)-glucan exposure that is distinct from that seen at bud scars, and is not based on increased numbers of bud scars.

The correlation between increased β (1,3)-glucan exposure and enhanced immune responses such as upregulated tumor necrosis factor alpha (TNF-α) secretion has been studied intensively [12–14, 17, 42, 43]. Exposed β (1,3)-glucan is recognized by the receptor Dectin-1 on the surface of immune cells including macrophages and neutrophils, and this recognition activates the host immune response for fungal clearance including the secretion of TNF-α [12]. To determine if the increased β (1,3)-glucan exposure in the *STE11Δ*^*N467*^ strain is immunologically relevant, we performed an enzyme-linked immunosorbent assay (ELISA) to quantify TNF- α secretion from RAW264.7 macrophages exposed to this strain. As seen in Fig 3D, TNF-α secretion was significantly upregulated when the Cek1 MAPK pathway was hyper-activated (*STE11Δ*^*N467*^ in YPM). It should be considered when examining the data in Fig 3D that production of TNF-α is reduced in all strains that were grown in YPM, including wild-type and *cho1ΔΔ*. Thus, while the increase in TNF-α of cultures of *STE11Δ*^*N467*^ grown in YPM is ~35% greater than that in YPD, the increase of *STE11Δ*^*N467*^ over wild-type, both grown in YPM, is 2-fold. Thus, increased β (1,3)-glucan exposure in the *STE11Δ*^*N467*^ strain increases pro-inflammatory responses from macrophages.

### *CHO1* disruption upregulates Cdc42 activity in *C*. *albicans*

**Fig 4:**
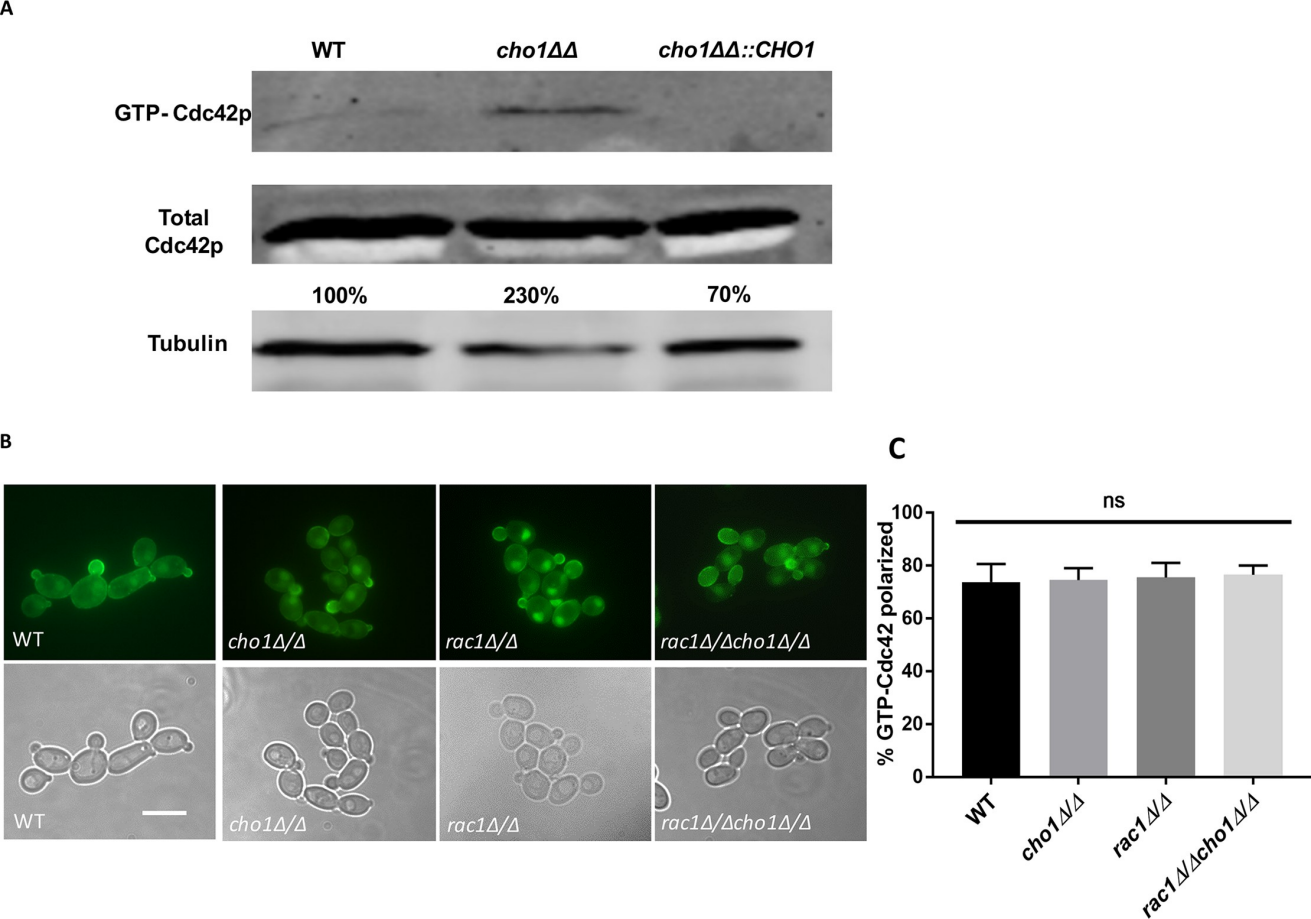
Cdc42 activity is upregulated in *cho1Δ/Δ* compared to wild-type. (A) GTP-Cdc42 was pulled-down with beads conjugated with GST-CRIB, which specifically binds with active GTP-Cdc42/Rac1. Cdc42 that was pulled down was then detected via Western blotting with anti-Cdc42 antibody. The amount of total Cdc42 in the extract was also probed as a control. The GTP-Cdc42/Total-Cdc42 ratio is expressed as a percentage of wild-type. (B) CaCRIB-GFP localization is not altered upon *CHO1* deletion. The CaCRIB-GFP probe was transformed into *Candida* strains to investigate the active GTP-Cdc42 localization. The scale bar represents 10μm. (C) Cells from Fig 4B were analyzed by microscopy for the number that exhibited CaCRIB-GFP localization to buds. Quantification is of three biological replicates, and each replicate has at least 50 cells.

The above results indicate that hyper-activation of Ste11 can cause unmasking, and since the Cek1 MAPK pathway, which acts downstream of Cdc42 [18], is constitutively activated in *cho1Δ/Δ*, this suggests that Cdc42 activity might be upregulated in *cho1Δ/Δ* (Fig 1). To test this possibility, Cdc42 activity was measured by monitoring the amount of active Cdc42 (GTP-bound) in cells. GTP-bound Cdc42 was isolated using agarose beads coated with Cdc42/Rac1 interactive binding (CRIB) domain [44]. As seen in Fig 4A, the concentration of GTP-bound Cdc42 in *cho1Δ/Δ* is higher than that in wild-type and *cho1ΔΔ*::*CHO1* strains. This confirms our hypothesis that *cho1Δ/Δ* has a higher concentration of active Cdc42 than wild-type. Thus, Cho1 or its biochemical product PS may impact Cdc42 activity negatively in wild-type cells, although this regulation may be indirect.

We then compared the localization of active Cdc42 in *cho1Δ/Δ* and wild-type by using a CaCRIB-GFP probe [44]. This motif binds with both Cdc42 and Rac1 GTPases. As seen in Fig 4B, both wild-type and *cho1Δ/Δ* cells have similarly localized active Cdc42, with the CRIB-GFP probe concentrated at the growth sites (buds). CRIB-GFP can bind both Rac1 and Cdc42, so to measure Cdc42 localization alone, we disrupted *RAC1* in both wild-type and *cho1Δ/Δ* by using a *C*. *albicans* CRISPR-Cas9 system [45]. Both *rac1Δ/Δ* and *cho1Δ/Δ rac1Δ/Δ* mutant cells have a similar pattern of CRIB-GFP localization during budding growth compared to wild-type and *cho1Δ/Δ* (Fig 4B and 4C). This suggests that active Cdc42 is found in its normal localization in *cho1Δ/Δ* cells.

**Fig 5:**
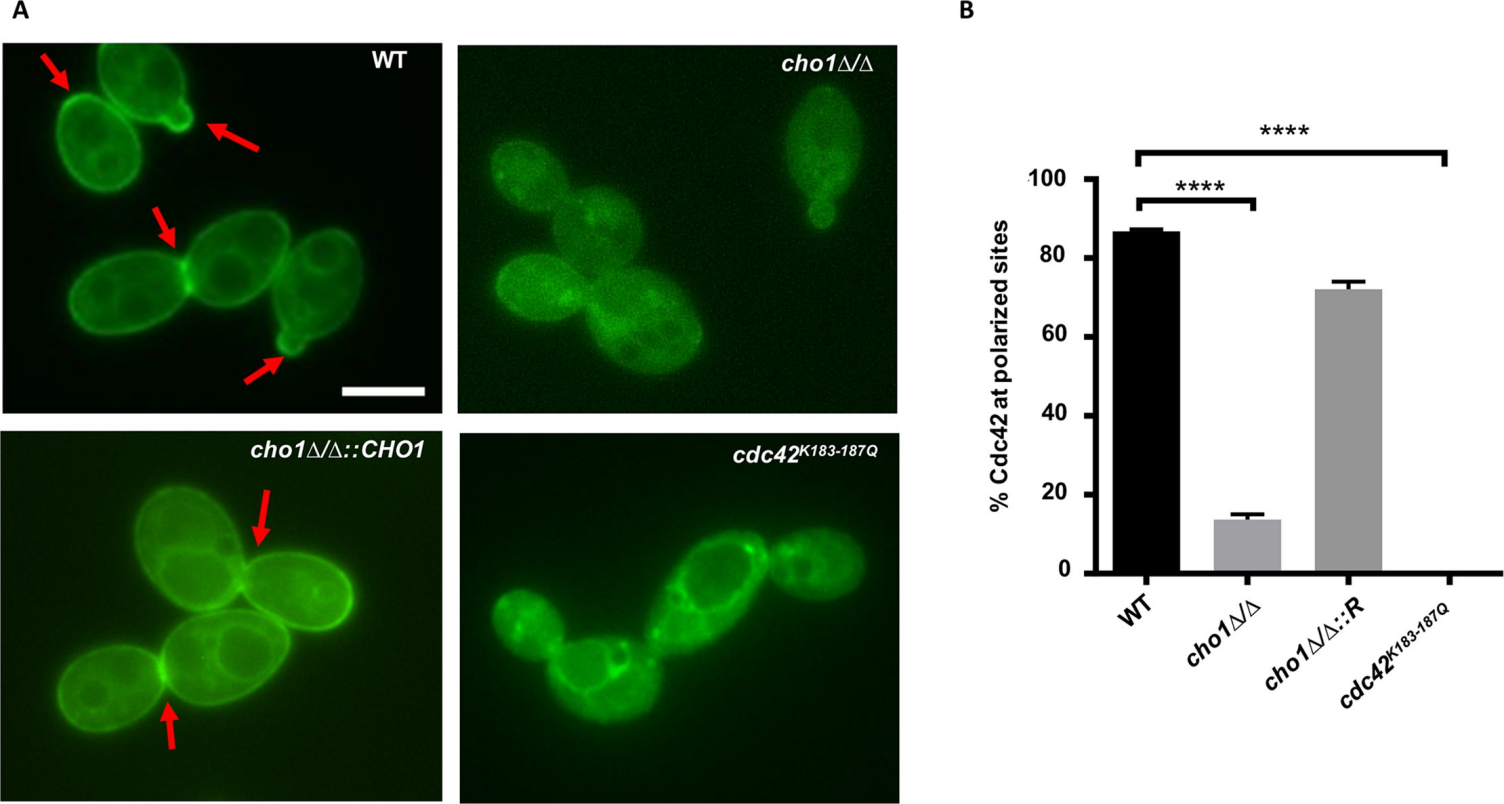
Cho1 is essential for GFP-Cdc42 polarization at the plasma membrane. (A) GFP-Cdc42 localization is examined for each strain by microscopy. The red arrows indicate the fluorescence concentrated at the bud tips or bud necks. The scale bar represents 5μm. (B) Quantification of the degree of polarization of GFP-Cdc42 in *Candida* cells. A minimum of 50 cells was counted for each strain and the imaging experiment was repeated three times. The statistical analysis was carried out by One-way ANOVA. ****, p<0.0001.

These results were in potential contrast to those for Cdc42 in a *S*. *cerevisiae cho1Δ* mutant, where PS disruption causes impaired Cdc42 polarization [46]. However, in this study, Fairn *et al*. used a GFP-Cdc42 construct to examine localization, which should visualize total Cdc42 rather than just active Cdc42. Therefore, we examined localization of total GFP-Cdc42 in *C*. *albicans cho1Δ/Δ* to determine how total Cdc42 responds to PS deficiency (Fig 5A). In the wild-type and reintegrated strains (*cho1Δ/Δ*::*CHO1*), Cdc42 is localized to the plasma membrane and internal membranes, and accumulates in bud necks and bud tips. The *cho1Δ/Δ* mutant has impaired polarization of GFP-Cdc42 to bud necks and tips. There is an overall decrease in plasma membrane binding of GFP-Cdc42, and instead GFP-Cdc42 accumulates in the cytoplasm. Approximately 80% of wild-type yeast cells have polarized Cdc42 localization, while only 20% of *cho1Δ/Δ* cells show polarized localization (Fig 5B). This result indicates that *CHO1* is necessary for the proper localization of total GFP-Cdc42 in *C*. *albicans*.

We next examined the mechanism by which PS may impact GFP-Cdc42 localization to buds and bud necks. PS is the most abundant anionic phospholipid of the plasma membrane, and it is largely restricted to the inner leaflet [46, 47]. A C-terminal polybasic region in some Rho-family small GTPases is a crucial domain for lipid interaction, where several positively charged amino acid residues promote plasma membrane localization, and have been suggested to do so via electrostatic interactions with negatively charged phospholipids including PS [48, 49]. To elucidate if this domain is crucial for localization of Cdc42 in *C*. *albicans*, we constructed a GFP-Cdc42 mutant where the four C-terminal lysines were mutated to glutamines (GFP-Cdc42^K183-187Q^), and observed its localization in *C*. *albicans* wild-type cells. As shown in Fig 5A, most of the GFP-Cdc42^K183-187Q^ was associated with endomembrane structures instead of the plasma membrane. Of note, the preferential accumulation of GFP-Cdc42 seen in the buds of normal wild-type yeast was absent in the mutated Cdc42^K183-187Q^ protein. This indicates that the C-terminal polybasic region of Cdc42p is important for association of total GFP-Cdc42 with plasma membrane. However, this does not show if the C-terminal domain is regulating localization by directly interacting with PS, although that is one possibility.

In contrast, as observed in Fig 4, GTP-bound unmodified Cdc42 is still able to associate with the bud necks and tips in the absence of *CHO1*, indicating that active Cdc42 can still localize to the appropriate places in the cell. The discrepancy we see between total GFP-Cdc42 localization and localization of GTP-bound native Cdc42 could be caused by the GFP or reflect differences between total versus active Cdc42 populations.

### Activation of Cdc42 contributes to ß(1,3)-glucan exposure

The above results indicate that the GTPase Cdc42 has increased activation in *cho1Δ/Δ* (Fig 4). To further investigate if this up-regulated Cdc42 activity contributes to β (1,3)-glucan exposure, we constructed a mutant strain that ectopically expresses a *CDC42* hyperactive allele (*CDC42*^*G12V*^) in wild-type. Introduction of *CDC42*^*G12V*^ decreases the intrinsic GTPase activity, therefore increasing the proportion of Cdc42 in an active GTP-bound state [18]. Cells overexpressing *CDC42*^*G12V*^ exhibited decreased proliferation in YPD liquid and poor growth on YPD agar plates [32]. Similarly, a hyper-activated *CDC42*^*G12V*^ mutant was dominant lethal in *S*. *cerevisiae* [50]. Our strain is viable, but does exhibit growth defects, so we measured β (1,3)-glucan exposure in the *CDC42*^*G12V*^ mutant by staining with anti-β (1,3)-glucan antibody, but also co-stained cells with propidium iodide to control for cell-viability. Propidium iodide staining revealed that the overnight *CDC42*^*G12V*^ culture contained fewer live cells compared to wild-type (S4A Fig). However, within the live cell populations for both strains, there was a much greater level of β (1,3)-glucan exposure in the *CDC42*^*G12V*^ cells compared to wild-type (S4B Fig). This suggests that increased Cdc42 activity causes β (1,3)-glucan exposure with the caveat that *CDC42*^*G12V*^ is clearly having pleiotropic effects.

### The Rho1-associated signaling pathway does not have a clear role in causing β(1,3)-glucan exposure

**Fig 6:**
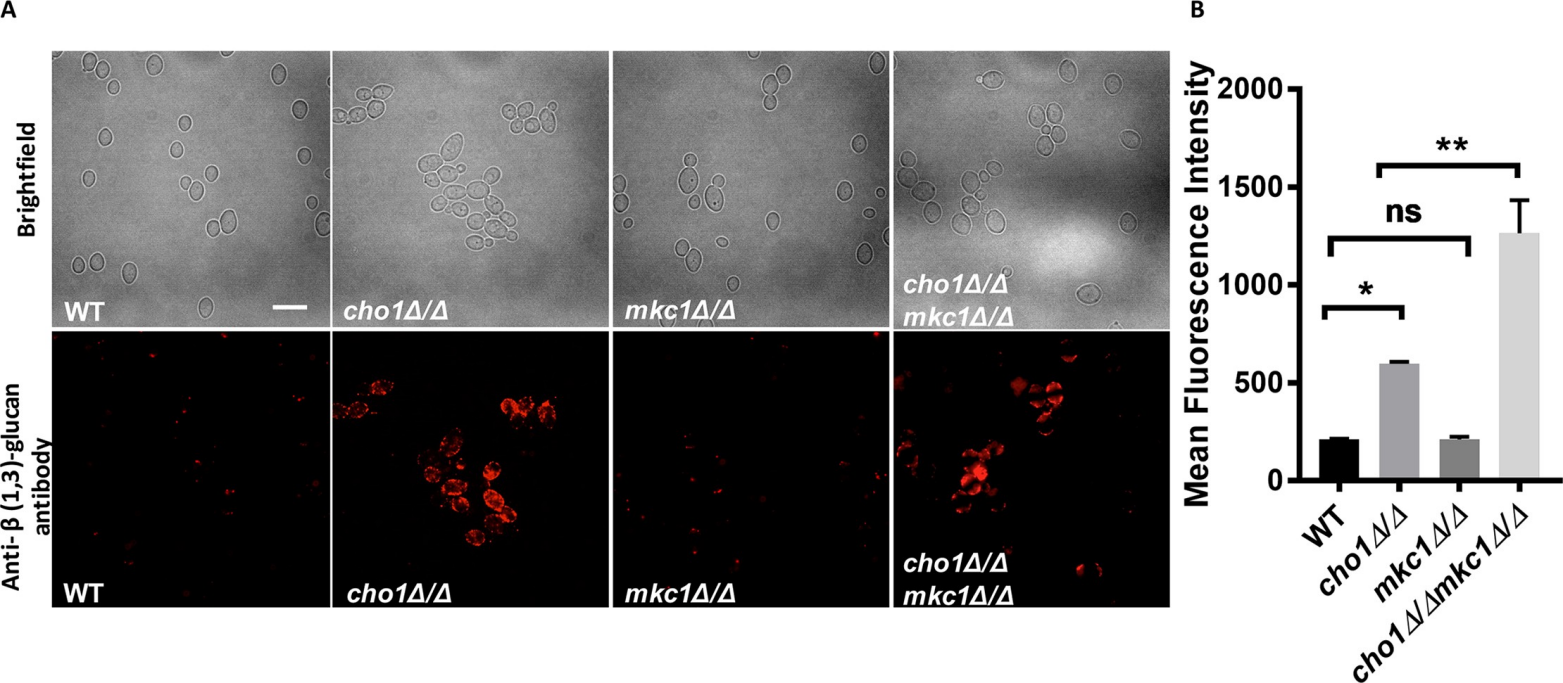
Deletion of *MKC1* in *cho1Δ/Δ* did not diminish β (1,3)-glucan exposure. (A) Cells were stained with primary anti-β (1,3)-glucan antibody and Cy3-conjugated secondary antibody, and imaged by epi-fluorescent microscopy. The scale bar indicates 10μm. (B) Flow cytometry was carried out to quantify β (1,3)-glucan exposure. Cells were incubated with primary anti-β (1,3)-glucan antibody and PE-conjugated secondary antibody. The statistical analysis was carried out by doing One-way ANOVA analysis. *, P = 0.0485; **, P = 0.0024.

Our data indicate that the Cek1 pathway can cause β(1,3)-glucan exposure when hyper-activated, and this may help explain the increased β(1,3)-glucan exposure seen in the *cho1Δ/Δ* mutant. However, the Mkc1 pathway is also upregulated in *cho1Δ/Δ* (Fig 2), and we wanted to determine if activation of this pathway plays a role in β(1,3)-glucan exposure as well. First, both *MKC1* alleles were disrupted via the *C*. *albicans* CRISPR-cas9 system [45] in wild-type and *cho1Δ/Δ*. Western blotting was performed to confirm that Mkc1 was not expressed in the mutants with both *MKC1* alleles disrupted (S5 Fig). Immunostaining with anti-β (1,3)-glucan antibody on wild-type, *cho1Δ/Δ*, *mkc1Δ/Δ* and *cho1Δ/Δ mkc1Δ/Δ* strains showed that deletion of *MKC1* did not rescue the β(1,3)-glucan exposure phenotype in the *cho1Δ/Δ* mutant (Fig 6). In fact, flow cytometry demonstrated that the *mkc1Δ/Δ cho1Δ/Δ* double mutant cells exhibited increased levels β (1,3)-glucan exposure compared to *cho1Δ/Δ* (Fig 6B). This suggests that Mkc1 MAPK probably plays a role in sustaining cell wall organization when *CHO1* is disrupted.

Pkc1 acts as a signaling module to connect Rho1 to the Mkc1 MAPK cascade [25–27]. We deleted one *PKC1* allele in *cho1Δ/Δ*, and this did not suppress the β(1,3)-glucan exposure phenotype (S6 Fig). Attempts to make a complete *cho1Δ/Δ pkc1Δ/Δ* double mutant failed. This does not completely test for a role for Pkc1 in unmasking, but is consistent with those above indicating that increased activation of the Mkc1 pathway does not cause β(1,3)-glucan exposure.

**Fig 7:**
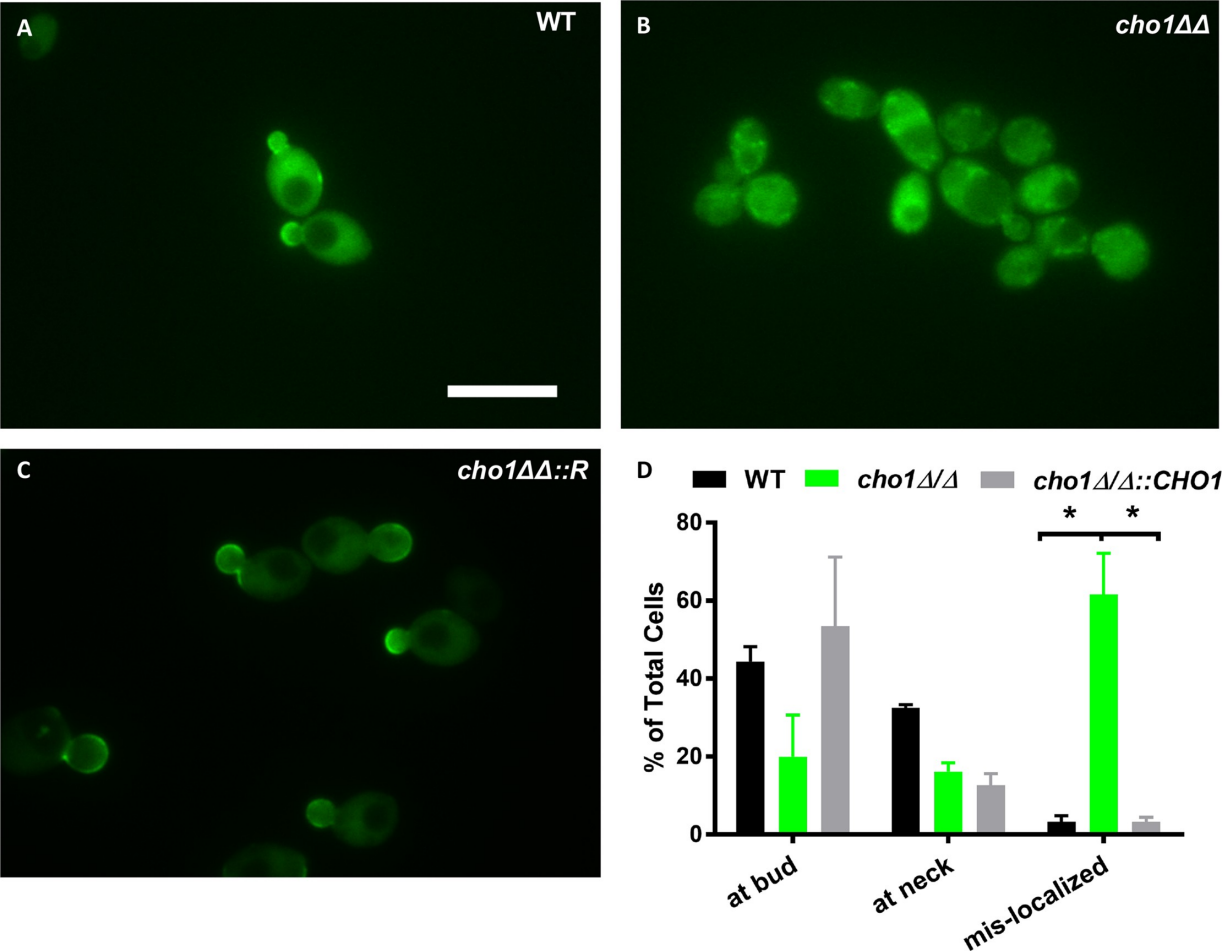
Active GTP-Rho1 is de-localized in *cho1Δ/Δ*. (A-C) GFP-RID localization was used as proxy for active (GTP-bound) Rho1 in cells, and was analyzed by epifluorescent microscopy. (D) Quantification of the degree of polarization of GTP-Rho1 in *Candida* cells. A minimum of 50 cells were counted for each strain and this repeated three times. The statistical analysis was done by One-way ANOVA. *, p<0.019.

**Fig 8:**
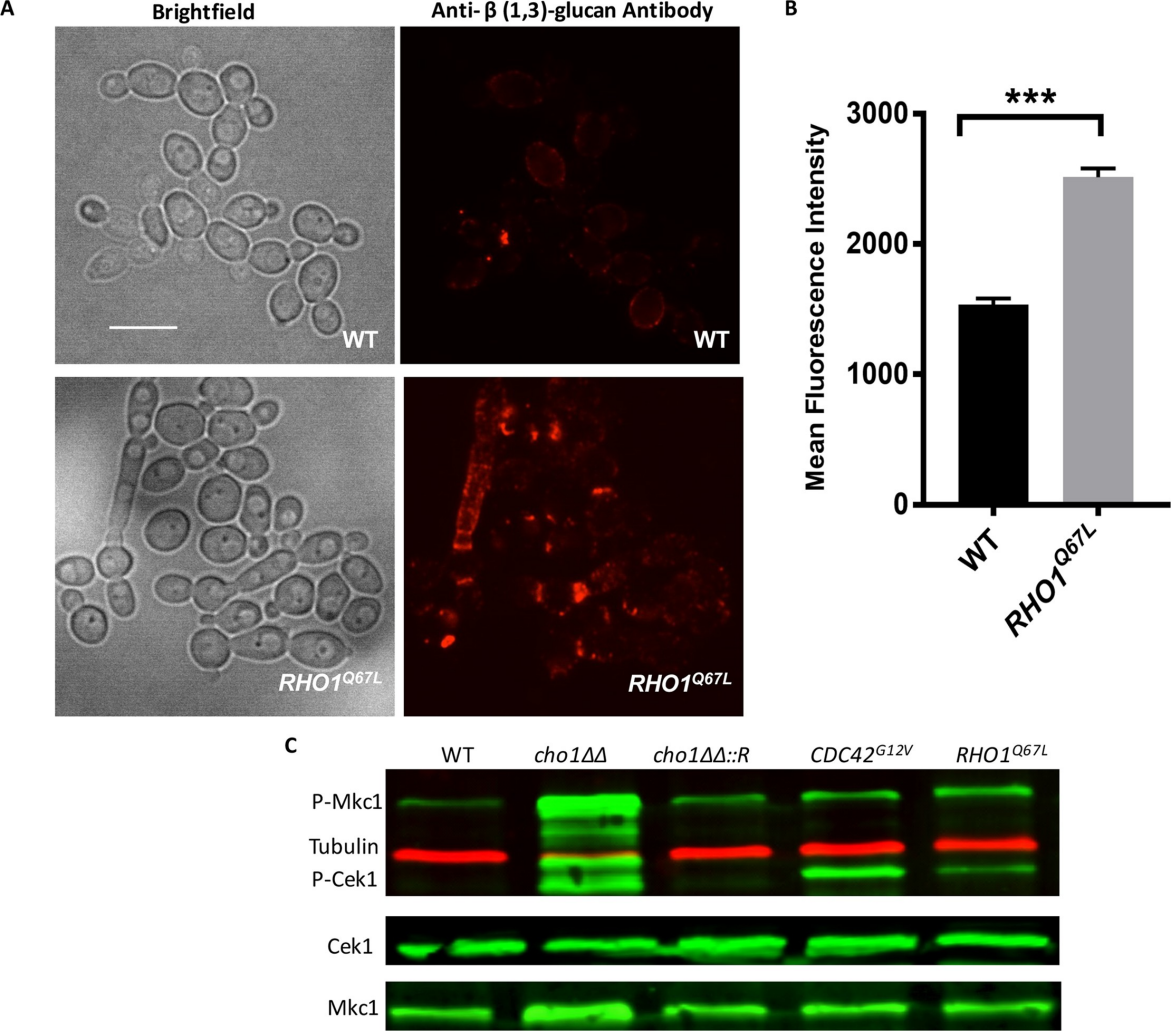
Hyper-activated Rho1 causes β (1,3)-glucan exposure. (A-B) *Candida* cells were stained for β (1,3)-glucan exposure as described in Fig 6. The scale bar represents 10μm. ***, p = 0.0003. (C) Western blotting was performed to examine the effect of expressing hyperactive *RHO1*^*Q67L*^ on the regulation of downstream MAPK activities. Phospho-p44/42 antibody was used to detect Phospho-Cek1 and Phospho-Mkc1 and anti-tubulin, anti-Mkc1, and anti-Cek1 antibodies were used as controls.

We then examined if Rho1 might play a role in increased β(1,3)-glucan exposure in *cho1Δ/Δ*. Total, but not active, Cdc42 is mislocalized in *cho1Δ/Δ* (Figs 4 and 5), therefore, we measured the distribution of active GTP-Rho1. This was achieved using a probe for active Rho1, that consists of a GFP tagged *C*. *albicans* Pkc1 Rho Interactive Domain (GFP-RID) [44]. In wild-type and *cho1Δ/Δ*::*CHO1*, GFP-RID is localized to the growth sites (i.e. buds and sites of cell division) (Fig 7), however the signal in *cho1Δ/Δ* is delocalized. This suggests that the Rho1 cell wall remodeling system might be re-localized when Cho1 is disrupted. Rho1 also has multiple lysines on its extreme C-terminus, similar to Cdc42, thus its mislocalization in *cho1Δ/Δ* could be affected for similar reasons as observed for total GFP-Cdc42 (Fig 5). Due to the lack of GFP-Rho1, we have not examined GFP-Rho1 to find the exact localization of total Rho1 in *cho1Δ/Δ*. The increased activation of Mkc1 in *cho1Δ/Δ* suggests that its upstream regulator, Rho1, might exhibit a similar increase in activation. To test if up-regulated Rho1 can cause β (1,3)-glucan exposure, we constructed a strain that ectopically expresses a hyperactive allele of *RHO1*^*Q67L*^ in wild-type. Introduction of the *RHO1*^*Q67L*^ allele decreases the ability of Rho1 to cleave GTP to GDP, therefore increasing the level of GTP-Rho1[25]. As shown in Fig 8A and 8B, hyper-activated *RHO1*^*Q67L*^ did cause a significant increase in cell wall unmasking compared to wild-type, but not as great as that seen with *STE11Δ*^*N467*^. However, examination of MAPK phosphorylation revealed that active Cek1 was unexpectedly upregulated along with active Mkc1 (Fig 8C). Thus, the β(1,3)-glucan exposure in the *RHO1*^*Q67L*^ strain could be due at least in part to Cek1 activation rather than Mkc1 (Fig 8C).

## Discussion

Previously, our lab showed that the enzyme for synthesizing PS, Cho1, plays a role in controlling β (1,3)-glucan exposure [13]. The homozygous PS synthase knockout mutant, *cho1Δ/Δ*, exhibits increased β (1,3)-glucan exposure compared to wild-type [13]. However, the mechanism by which *cho1Δ/Δ* displays the β (1,3)-glucan exposure phenotype was unclear.

In this report, we identify two MAPK signaling pathways (Cek1 and Mkc1) that are activated in the *cho1Δ/Δ* mutant (Fig 2), and we hypothesized that one or both may contribute to increased β(1,3)-glucan exposure in *cho1Δ/Δ*. MAPK signal transduction cascades are essential pathways for *C*. *albicans’* adaptation to the host environment [35, 51]. Cek1 and Mkc1 are major MAPK pathways in this organism that play roles in cell wall regulation. The Mkc1-associated pathway is primarily responsible for cell wall integrity, while the Cek1-mediated signaling cascade is important for cell wall construction and hyphal formation [19, 52–54].

### Cdc42-Cek1 MAPK pathway activation can increase β(1,3)-glucan exposure

We tested the hypothesis that one or both of these pathways can cause β (1,3)-glucan exposure in *cho1Δ/Δ* by determining if they could contribute to this phenotype independently of loss of PS. We found confirming evidence for the Cek1 pathway. In particular, a hyperactive form of Ste11 (*STE11*^*ΔN467*^), the MAPKKK that activates Cek1 (Fig 1), stimulates significant β (1,3)-glucan exposure in yeast-form cells compared to wild-type cells (Fig 3). This confirms an assertion that β (1,3)-glucan can be unmasked in Cek1 inducing conditions [39]. The cells with *STE11Δ*^*N467*^*-*induced unmasking also exhibit more TNF-α secretion from macrophages (Fig 3D).

Ste11 is downstream of the small GTPase Cdc42 (Fig 1), which has been well-studied in *C*. *albicans* [18, 32, 44, 55]. Cdc42 is involved in cellular proliferation and bud emergence and activates the downstream protein kinase Cst20, which also controls the activation of the Cek1 MAPK cascade including Ste11 [15, 18, 19, 52]. To control accurate cellular function, Cdc42 cycles between an active GTP-bound and inactive GDP-bound state [55]. By performing pull-downs of GTP-Cdc42 with CaCRIB-GST and Western blotting, we have evidence that the level of active GTP-Cdc42 is higher in *cho1Δ/Δ* compared to wild-type (Fig 4A). This might be responsible for the increased activation of the downstream Ste11-associated cascade. We did not test to see if disruption of *CEK1* in the *cho1Δ/Δ* strain would decrease β (1,3)-glucan exposure because *cek1Δ/Δ* also exhibits more exposed β(1,3)-glucan than wild-type [15], and this would be uninterpretable.

### Possible roles of PS in regulating Cdc42 activity and localization

The impact of PS on β (1,3)-glucan exposure is likely indirect, but may be occurring through its role in regulating Cdc42. The loss of PS correlates with increased Cdc42 activity, which in turn can lead to activation of the Cek1 pathway, which does cause β (1,3)-glucan exposure when activated (Fig 3). However, the mechanism by which loss of PS causes Cdc42 activation is currently unclear, but possibilities are discussed below.

PS may impact Cdc42 activity indirectly by regulating the GTPase activating proteins (GAPs) for Cdc42. These GAPs act as repressors of Cdc42 activity. Previous investigations identified that PS stimulates the GAP activity of Rga1 and Rga2 toward Cdc42 in *S*. *cerevisiae* [56]. Given that *C*. *albicans cho1Δ/Δ* lacks PS [16], this may result in less inhibition of the GAP activity, and in turn results in less inhibition of Cdc42 activity.

In addition, there are data indicating that PS can control the localization of a subpopulation of Cdc42. For example, we found that GFP-tagged Cdc42 is mislocalized in *C*. *albicans cho1Δ/Δ*. Moreover, mutating the C-terminal lysines to glutamine in GFP-Cdc42 led to mislocalization of GFP-Cdc42^K183-187Q^ in wild-type cells (Fig 5). This is similar to what has been observed in *S*. *cerevisiae*, where Cdc42 localization is affected by both PS and the basic lysine residues at the C-terminal domain of Cdc42 [46, 49]. However, in contrast to this, the localization of active GTP-Cdc42 in *C*. *albicans*, as measured by CaCRIB-GFP (binds to GTP-Cdc42/Rac1) appears to be focused in the bud necks and tips like wild-type (Fig 4B). Therefore, PS might control only a subpopulation of Cdc42 localization. It is also possible that GFP-Cdc42 does not fully represent endogenous Cdc42 in its activated state.

The mechanism by which PS controls Cdc42 localization in *C*. *albicans* remains to be fully elucidated. One model suggests that Cdc42 localization is controlled in part through the interaction between the negatively charged PS head group and the lysines at the C-terminus of Cdc42 (Fig 5). However, this is only a model at this point and remains to be tested, as the impact of PS on GFP-Cdc42 may be indirect. These lysines may interact with another protein that is required to localize Cdc42 that itself is impacted by PS. In addition, the correct localization of active Cdc42 in *cho1Δ/Δ* indicates that other factors, perhaps GEFs or GAPs, play an important role in Cdc42 localization, independently of PS (Fig 4).

### Activation of the Mkc1 MAPK pathway does not appear to be sufficient to cause unmasking

The other MAPK pathway upregulated in *cho1Δ/Δ* is the Mkc1 pathway (Figs 1 and 2). We tested for its role in *cho1Δ/Δ*-dependent β (1,3)-glucan exposure by generating a *cho1Δ/Δ mkc1Δ/Δ* double mutant, and this did not diminish β(1,3)-glucan exposure (Fig 6). Moreover, we disrupted one allele of the upstream kinase Pkc1, and this also did not diminish β (1,3)-glucan exposure (S6 Fig). Finally, a hyperactive GTP-bound form of Rho1 (*RHO1*^*Q67L*^) was generated, and it did lead to modest β (1,3)-glucan exposure compared to wild-type, however surprisingly it also led to increased phosphorylation of Cek1 as well as Mkc1 (Fig 8), thus the increase may be caused by Cek1 upregulation.

An alternative role for Mkc1 may be to diminish β (1,3)-glucan exposure in stress conditions. For example, the *mkc1Δ/Δ* mutant did not exhibit enhanced β (1,3)-glucan exposure compared to wild-type, but the *cho1Δ/Δ mkc1Δ/Δ* double mutant exhibited greater β (1,3)-glucan exposure than *cho1Δ/Δ* alone. This, coupled with the mislocalization of active Rho1 in *cho1Δ/Δ* (Fig 7), may indicate that Mkc1 is activated to compensate for cell wall disfunction that is caused by the *cho1Δ/Δ* mutation, perhaps even due to upregulated Cek1.

Surprisingly, our results with the Mkc1 pathway’s relationship to PS contrast with what is observed for the orthologous pathway in *S*. *cerevisiae*. In baker’s yeast, PS has been shown to be necessary for the activation of the *S*. *cerevisiae* Mkc1 homolog Slt2 [22, 57]. However, we observed that loss of PS synthase in *C*. *albicans* causes increased Mkc1 activity (Fig 2), suggesting that there are fundamental differences in the manner through which the Mkc1-associated cascade is regulated in pathogenic versus non-pathogenic yeasts. This report also sets the stage for better understanding how the phospholipid PS synthase influences GTPase activity and localization in this pathogenic organism.

### Conclusions

*Candida albicans* is able to diminish its detection by innate immune cells through masking of β (1,3)-glucan in the inner cell wall with an outer layer of heavily glycosylated mannoproteins (mannan)[12, 58, 59]. Once exposed, this glucose polymer antigen can be detected by Dectin-1, a C-type signaling lectin found on host immune cells [10, 11]. However, it usually takes several days after infection before β (1,3)-glucan is exposed to the immune system [58]. Therefore, if the β (1,3)-glucan exposure process could be induced more rapidly, the immune responses would be expected to improve and clear fungal pathogens more effectively [12, 58, 60, 61].

Identification of specific pathways that contribute to β (1,3)-glucan exposure when activated could help elucidate future drug targets that can induce β (1,3)-glucan exposure to improve immune response. Thus, compounds that specifically activate Cek1 may be useful in this regard. If such compounds were combined with the current azole class of antifungals, which act statically, and immune detection were simultaneously enhanced, this could potentially enhance the clearance of fungi.

## Methods

### Strains and growth media

All of the strains and plasmids used for these experiments are described in S1 and S2 Tables. The medium used to culture strains was yeast extract-peptone-dextrose (YPD) medium (1% yeast extract, 2% peptone, and 2% dextrose (Thermo Fisher Scientific) (unless otherwise stated) [62]. To express the gene from the promoter of ATP sulfurylase (*MET3*), SD minimal medium (2% dextrose, 0.67% Yeast nitrogen base without amino acids) with 1mM ethanolamine (to support *cho1Δ/Δ*) was used[63]. For the induction of genes under the control of the *MAL2* maltase promoter, YPM (1% yeast extract, 2% peptone, and 2% maltose, Thermo Fisher Scientific)[64] was used. To induce hyphal formation, cells were sub-cultured in Gibco RPMI 1640 medium (Thermo Fisher Scientific).

### Strain construction

Plasmid construction is described in S1 Text and plasmids used in this report are listed in S2 Table. Primers used in this study are listed in S3 Table.

### Western blotting

Cells were grown overnight in liquid YPD at 30°C, diluted to an OD_600_ of 0.2 in fresh YPD medium and allowed to grow for 3 hours. For the *STE11*^*ΔN467*^ strain under the *MAL2* promoter, cells were grown overnight in liquid YPM at 30°C, and diluted back to OD_600_ of 0.1 into fresh YPM medium and grown to log phase. Cells were pelleted by centrifugation, and resuspended in 250μl phosphate buffered saline (PBS) supplemented with protease inhibitor cocktail (PMSF, leupeptin, and pepstatin (RPI, Corp., Mount Prospect), complete Protease Inhibitor tablet and PhosStop Phosphatase Inhibitor tablet (Roche Diagnostics GmbH, Mannheim, Germany). An equal volume of 150–212μm acid-washed beads (Sigma Aldrich, MO, USA) was added to each tube. Cells were mechanically disrupted in a Biospec Mini-BeadBeater (Bio Spec Product Inc., USA) with 6 rounds of 1min homogenization at 4°C and 1min intervals for each cycle. Samples were centrifuged at 5,000×rpm for 10 min at 4°C, the supernatant was collected, and the protein concentration was quantified using the Bradford protein assay (Bio-Rad Laboratories Inc., USA). Extracts were heated for 3 min at 95°C, and equal amounts of protein from each sample were separated on an SDS-12% polyacrylamide gel. Separated proteins were transferred onto a polyvinylidene difluoride (PVDF) membrane with a Hoefer MiniVE vertical electrophoresis unit (Amersham Biosciences Inc., USA). Membranes were blocked in blocking buffer (LI-COR biosciences Inc., USA) at room temperature for 1hour and subsequently incubated overnight at 4°C with Anti-phospho-p44/p42 MAPK (Thr202/Tyr204) antibody at a 1:2000 dilution (Cell Signaling Technology, Inc., USA) to detect phosphorylated Mkc1 and Cek1 MAPKs. The expression of total Mkc1 was detected with the primary antibody against total Mkc1 (1:1000). The expression of total Cek1 was measured with an antibody to total Cek1 (1:1000). The secondary antibody against Phospho-p44/42 Ab, Mkc1 Ab and Cek1 Ab was IRye800CW goat anti–rabbit IgG (H+L) conjugate (green, 1:10,000 dilution; LI-COR Biosciences) incubated in the dark followed by extensive washing and quantitation using an Odyssey IR imaging system (LI-COR Biosciences). Phosphorylated and total proteins levels were quantitated using ImageJ (National Institutes of Health, Bethesda, MD). As a control protein, tubulin was detected with rat anti-tubulin primary antibody (Bio-Rad Laboratories Inc., USA) at a 1:1000 dilution and IRDye 680RD Goat-anti-Rat IgG (H+L) (red, 1:10,000 dilution; LI-COR Biosciences).

### Pull-down assay for active Cdc42

Cells were grown in YPD to log phase, and pelleted by centrifugation, and re-suspended in Lysis/Binding/Wash buffer, provided by Active Cdc42 Pull-Down and Detection Kit (Thermo Fisher Scientific) with protease inhibitors cocktail (PMSF, leupeptin, and pepstatin) (RPI, Corp., Mount Prospect) and complete phosphatase inhibitor tablet (Roche Diagnostics GmbH, Mannheim, Germany), and cells were disrupted with acid-washed glass beads (Sigma-Aldrich Co. LLC., USA) in a Biospec Mini-Bead Beater with 6 rounds of 1min homogenization at 4°C and 1min interval for each cycle. The protein concentration was quantified using the Bradford protein assay (Bio-Rad Laboratories Inc., USA).

1,500 μg of total protein were used for the pull-down procedure following the instruction from Active Cdc42 Pull-Down and Detection Kit (Thermo Fisher Scientific). 50ul of the pull-down samples containing active Cdc42 were separated by SDS-PAGE, transferred to PVDF with the Hoefer MiniVE vertical electrophoresis unit (Amersham Biosciences Inc., USA), and detected with mouse monoclonal anti-Cdc42 antibody at a 1:250 dilution (Cytoskeleton Inc., USA), followed by secondary detection with IRye800CW goat anti–mouse IgG (H+L) conjugate (1:10,000; LI-COR biosciences). As a control protein, tubulin was detected with rat anti-tubulin primary antibody (Bio-Rad Laboratories Inc., USA) and IRDye 680RD Goat-anti-Rat IgG (H+L) (LI-COR biosciences). Densitometry quantification of Cdc42 bands was performed with ImageJ (National Institutes of Health, Bethesda, MD).

### Immunofluorescent imaging of β (1,3)-glucan exposure

This procedure was done as described in [13] with minor modification. *C*. *albicans* cells were grown overnight in YPD or YPM medium at 30°C. Mouse anti-β (1,3)-glucan antibody (Biosupplies Australia Pty Ltd., Australia) at a 1:800 dilution was used as the primary antibody, and a goat anti-mouse antibody conjugated to Cy3 (Jackson ImmunoResearch Inc., USA) at 1:300 dilution was used as secondary antibody. For imaging, *Candida* cells were resuspended in 100 μL of PBS and visualized with LEICA DM5500B epi-fluorescent microscope with Hamamatsu Orca-ER CCD digital camera (Model#C4742-80-12AG). The pictures were taken through Leica Application Suite AF (Advanced Fluorescence) software.

### Fluorescence Imaging

For imaging GFP-Cdc42 expressed under the *MET3* promoter, *Candida* cells were cultured overnight in SD minimal medium plus 1mM ethanolamine at 30°C, diluted to an OD_600_ of 0.2 in the fresh SD medium and allowed to grow for about 4–5 hours to reach the OD_600_ of 0.6–0.8. Cells carrying the CRIB-GFP or GFP-RID constructs (the expression of each is under the constitutive *ADH1* and *ACT1* promoters, respectively), were cultured in YPD medium. The overnight culture at 30°C was diluted back to an OD_600_ of 0.2 in fresh YPD medium and grown for 3 hours to reach log phase. 1mL of cells was collected and re-suspended in 100μl of PBS. 3μl of samples were mounted on the slide and observed under Leica DM RXA epi-fluorescent microscope with Leica DFC365FX CCD camera (Vashaw Scientific, Inc.). The pictures were taken through Leica Application Suite (LAS) V4.4 software.

### Flow cytometry

To stain the *STE11*^*ΔN467*^ strain (*P*_*MAL*_ promoter) and its controls, overnight cultures in YPM or YPD were collected and blocked in PBS plus 3% bovine serum albumin (BSA, Thermo Fisher Scientific, USA) for 30mins. Primary and secondary antibody incubations occurred on ice in PBS plus 3% BSA for 1.5 h and 20mins, respectively. Soluble Dectin-1–Fc (sDectin-1-Fc) [8] at 16.5 μg/ml was used to detect exposed β (1,3) glucan and mouse anti-Als3 antibody with 1:800 dilution was used for staining Als3 on hyphal cells. The Donkey anti-human IgG (H+L) Alexa Fluor 488 (Jackson ImmunoResearch) and goat anti-mouse antibody conjugated to R-Phycoerythrin (R-PE) were used as secondary antibodies, respectively.

To stain exposed β (1,3)-glucan on *CDC42*^*G12V*^ cells, overnight cultures were collected, and mouse anti-β (1,3)-glucan antibody at a 1:800 dilution and rabbit anti-mouse IgG (H+L) Alexa Fluor 488 (Jackson Immuno Research) were utilized as primary and secondary antibodies, respectively. 5ul of eBioscience^TM^ propidium iodide dye (Thermos fisher) was then added to the solution for the live/dead staining, and incubated for 5min at room temperature.

To stain β(1,3)-glucan in *Candida* cells with *MKC1* deleted, the overnight culture was incubated with mouse anti-β (1,3)-glucan antibody at a 1:800 dilution as primary antibody, and followed by goat anti-mouse antibody conjugated to R-Phycoerythrin (R-PE) at 1:300 dilution (Jackson ImmunoResearch) as a secondary antibody. The staining process for *RHO1*^*Q67L*^ strains was the same except that the overnight cultures were diluted back to OD_600_ at 0.1 and the log phage cells were collected after 3hrs growth for staining.

For all of the above conditions, after staining, cells were processed by washing five times with PBS, and samples were resuspended in 500μl of FACS buffer (PBS, 1% serum, 0.1% sodium azide) for flowcytometry in a FACSCalibur LSR II flow cytometer (Becton Dickinson). Singlets were gated by using a forward scatter area (FSC-A) versus side scatter area (SSA) plot, followed by forward scatter width (FSC-W) versus forward scatter area (FSC-A) density plot, as well as a side scatter width (SSC-W) versus side scatter area (SSC-A) plot to exclude clumping cells. We further compare the PE fluorescence intensity from the P3 singlets population in different *Candida* strains. Flow cytometry data were obtained for 100,000 gated events per strain and experiments were performed in triplicate, and analyzed using FlowJo software package with version 10.11 (FlowJo LLC, OR, USA).

### Enzyme-linked immunosorbent assay (ELISA) of TNF-α

RAW264.7 macrophages were plated the day prior at 5×10^5^/well in a 24-well plate. To activate *STE11*^*ΔN467*^ expression under *P*_*MAL*_ regulation, *STE11*^*ΔN467*^ mutant cells were grown in YPM. Overnight cultures were washed and diluted to an OD_600_ of 1.25 in 5ml PBS/well in a 6-well plate for UV-kill. To do this, the 6-well plate was placed in the Spectrolinker XL-1000 UV Crosslinker (Spectroline Inc., USA) and the ENERGY mode was set to 100,000 μJ/cm^2^. The UV-killing process was repeated 5 times. UV-killed *Candida* cells were then added to the RAW264.7 macrophages and coincubated at a 1:10 ratio for 4 h at 37°C and 5% CO_2_. The supernatant of each well was collected and filtered through a syringe filter with 0.2μm pore size (Millipore Sigma, US) to exclude the macrophage debris. The ELISA kit instructions from the manufacturer (R&D Systems) were followed. Each sample has three individual wells, and the statistical analysis was performed by using Two-way analysis of variance ANOVA (GraphPad Prism, v7.04 software).

## Supporting information

S1 Table *C*. *albicans* strains used in this study. (DOCX)

S2 Table Plasmids used in this study. (DOCX)

S3 Table Primers used in this study. (XLSX)

S1 Text Plasmid and Strain Construction. (DOCX)

S1 Fig The *STE11*^*ΔN467*^ strain exhibits significantly increased β (1,3)-glucan exposure compared to wild-type. Overnight cultures of *Candida* cells was incubated with anti-β (1,3)-glucan primary antibody and PE-conjugated secondary antibody, followed by flow cytometry to quantify the fluorescence intensity. Data represent three biological replicates. The statistical analysis was done by One-way ANOVA. ***, P = 0.0004; *, p = 0.0137
(TIF)

S2 Fig Growth curves were measured to determine the growth rate of strains in YPD vs YPM. Cells were grown overnight in YPD, diluted back to 0.1 OD600 and transferred to fresh YPD or YPM. A growth curve was performed with three replicates per condition, and plotted based on the growth rate of different strains measured in 48 hrs. The growth at each time-point between YPD and YPM cultures of *STE11*^*ΔN467*^ were compared by Two-way ANOVA(****, p<0.0001; *, p = 0.0286). The same comparison was made between wild-type YPD and YPM culture (####, p<0.0001; ###, p = 0.0007).
(TIF)

S3 Fig The exposed β (1,3)-glucan in *STE11*^*ΔN467*^ YPM cells was not restricted to bud scars. Overnight cultures of wild-type and *STE11*^*ΔN467*^ grown in YPM were co-stained with anti-β(1,3)-glucan antibody and Cy3 secondary to visualize exposed β(1,3)-glucan and calcofluor white to visualize chitin.
(TIF)

S4 Fig *CDC42*^*G12V*^ increases β (1,3)-glucan exposure, but also reduces the viable cell population. (A) Propidium iodide staining was performed to quantify the live cells in *Candida* strains. (B) β (1,3)-glucan exposure in live (gated for propidim iodide negative cells) wild-type and *CDC42*^*G12V*^ populations was measured by flow cytometry.
(TIF)

S5 Fig *MKC1* was knocked out in *C*. *albicans* via CRISPR-Cas9. Western blotting was performed using anti-Mkc1 antibody to confirm the absence of Mkc1 in the *MKC1* knockout mutants compared to wild-type (WT) and other strains. Tubulin was probed with anti-tubulin antibody as a loading control.
(TIF)

S6 Fig Deleting one *PKC1* allele in *cho1Δ/Δ* did not rescue β (1,3)-glucan exposure. One *PKC1* allele was deleted by the SAT1-flipper method. Cells were then stained with anti-β (1,3)-glucan primary antibody and phycoerythrin (PE)-conjugated secondary antibody. The statistical analysis was carried out by doing One-way ANOVA.
(TIF)
