# Supplementary material for: Exposure of Candida albicans β (1,3)-glucan is promoted by activation of the Cek1 pathway
Source: PLoS Genet. 2019 Jan 31;15(1):e1007892. doi: 10.1371/journal.pgen.1007892 (PMC6372213; doi:10.1371/journal.pgen.1007892)
Supplement: S1 Table — (DOCX) [file pgen.1007892.s001.docx]

**S1 Table. *C. albicans* strains used in this study**

| Strain | Parent | Genotype | Source or reference |
| --- | --- | --- | --- |
| SC5314 | Clinical isolate | Prototrophic wild type | Gillum et al, 1984 |
| YLC337 | SC5314 | *cho1Δ/Δ* | [16] |
| SED022 | YLC337 | *cho1Δ/Δ::CHO1* | [13] |
| YLC375 | SC5314 | *psd1Δ/Δ psd2Δ/Δ* | [16] |
| TC001 | SC5314 | Wild-type *P_MET3_-GFP-CDC42* | This study |
| TC002 | YLC337 | *cho1Δ/Δ P_MET3_-GFP-CDC42* | This study |
| TC003 | SED022 | *cho1Δ/Δ::CHO1 P_MET3_-GFP-CDC42* | This study |
| TC004 | YLC375 | *psd1Δ/Δpsd2Δ/Δ P_MET3_-GFP-CDC42* | This study |
| TC005 | SC5314 | *mkc1Δ/Δ* | This study |
| TC006 | SC5314 | Wild-type *P_MET3_-GFP-CDC42^K183-187Q^* | This study |
| TC007 | SC5314 | *rac1Δ/Δ* | This study |
| TC011 | SC5314 | Wild-type *P_MAL_-STE11^ΔN467^* | This study |
| TC033 | YLC337 | *cho1Δ/Δrac1Δ/Δ* | This study |
| TC034 | YLC337 | *cho1Δ/Δmkc1Δ/Δ* | This study |
| TC046 | SC5314 | Wild-type *GFP-RID* | This study |
| TC064 | TC033 | *cho1Δ/Δrac1Δ/Δ* CRIBGFP | This study |
| TC071 | YLC337 | *cho1Δ/Δ GFPRID* | This study |
| TC072 | SED022 | *cho1Δ/Δ::CHO1* GFPRID | This study |
| TC073 | TC007 | *rac1Δ/Δ* CRIBGFP | This study |
| TC078 | SC5314 | Wild-type *P_ENO1_-CDC42^G12V^* | This study |
| TC083 | SC5314 | Wild-type *P_ENO1_-RHO1^Q67L^* | This study |
| TC107 | SC5314 | Wild-type *PKC1/pkc1Δ* | This study |
| TC110 | YLC337 | *cho1Δ/cho1Δ PKC1/pkc1Δ* | This study |
