# Supplementary material for: Exposure of Candida albicans β (1,3)-glucan is promoted by activation of the Cek1 pathway
Source: PLoS Genet. 2019 Jan 31;15(1):e1007892. doi: 10.1371/journal.pgen.1007892 (PMC6372213; doi:10.1371/journal.pgen.1007892)
Supplement: S2 Table — (DOCX) [file pgen.1007892.s002.docx]

**S2 Table. Plasmids used in this study**

| Strain Name | Description | Source |
| --- | --- | --- |
| pYLC314 | *CaNAT-P_MET3,_ ampR* | This study |
| pTC001 | *CaNAT-P_MET3_-GFP-CDC42, ampR,* | This study |
| pYLC146 | FLP-CaNAT, *chloraR* | This study |
| pBT1 | *CaNAT-P_ENO1_*, *ampR* | This study |
| pExpArg-pADH1CRIBGFP | pExpArg-pADH1CRIBGFP | [44] |
| pTC009 | pExpArg-pADH1CRIBGFP+HYG^R^ | This study |
| pTC014 | *CaNAT-P_MET3_-GFP-CDC42^K183-187Q^, ampR* | This study |
| pV1393 | *FLP-CAS9-CaNAT-SNR52p-sgRNA*, *ampR* | [45] |
| pTC015 | pV1393 *CaMKC1-sgRNA, ampR* | This study |
| pTC018 | pV1393 *CaRAC1*-*sgRNA*, *ampR* | This study |
| pTC019 | CaNAT-*P_MA_*_L_, *ampR,* | This study |
| pTC020 | pTC019 *CaSTE11^ΔN467^,* ampR | This study |
| pTC029 | pExpArg-pADH1CRIBGFP+Hyg^R^ | This study |
| pExpArg-pACT1GFPRID | pExpArg-pACT1GFPRID, *ampR* | [44] |
| pTC033 | pExpArg-pACT1GFPRID+NAT^R^ | This study |
| pTC034 | pBT1 *CaCDC42*, *ampR* | This study |
| pTC035 | pBT1 *CaRHO1*, *ampR* | This study |
| pTC037 | pBT1 *CaCDC42*^G12V^, *ampR* | This study |
| pTC038 | pBT1 *CaRHO1*^Q67L^, *ampR* | This study |
| pTC041 | pYLC146 CaPKC1-KO, *chloraR* | This study |
