# Supplementary material for: Exposure of Candida albicans β (1,3)-glucan is promoted by activation of the Cek1 pathway
Source: PLoS Genet. 2019 Jan 31;15(1):e1007892. doi: 10.1371/journal.pgen.1007892 (PMC6372213; doi:10.1371/journal.pgen.1007892)
Supplement: S1 Text — (DOCX) [file pgen.1007892.s004.docx]

**S1 Text-Plasmid and Strain Construction**

For the generation of strains that conditionally express green fluorescent protein (GFP) tagged Cdc42 under the regulation of *P_MET3_*, we used the plasmid pTC1 composed of *P_MET3_*-*yEGFP*-*CDC42*, which was constructed as follows. *yEGFP* was amplified with primers TRO988+TRO989 with *BamHI* flanking both ends, and *CDC42* was amplified from SC5314 genomic DNA with primers TRO995+TRO996 with *SacI* flanking both ends. Both fragments were cloned into pYLC314 (Tams et al, In Press) 3’ to the *MET3* promoter, where *yEGFP* is in frame and 5’ to *CDC42* and connected by a sequence that encodes an 8 alanine linker. *CDC42^K183-187Q^* was chemically synthesized by Genescript Inc. with SacI sites. It was used to replace wild-type *CDC42* in pTC1 to create pTC14. Both pTC1 and pTC14 were linearized within *P_MET3_* using *AflII*, and then transformed into the strains of interests by electroporation. The transformation methods have been previously described (DeBacker et al, 1999). TCO1+TCO2 were used to validate if the fragment was integrated into *P_MET3_* locus in the chromosome.

To generate the strain CaTC11 that conditionally expresses *STE11^ΔN467^* under the regulation of *MAL2* promoter, we constructed the plasmid pTC20 that consists of *P_MAL2_*- *STE11^ΔN467^*-*T_TEF3_*. First, pTC19 containing the *P_MAL2_* promoter was generated from pYLC314. The *P_MET3_* was removed from pYLC314 by restriction with *PstI*. The regulatable promoter *P_MAL2_* was amplified from wild-type *C. albicans* genomic DNA using the primers TCO36+TCO37. The promoter was then cloned into the *PstI* site of the linearized pYLC314 to create pTC19. Then, the hyperactive domain of *STE11^ΔN467^* with a *TEF3* terminator was amplified from the *P_TET-ON_*- *STE11^ΔN467^* plasmid (generously provided by Dr. Joachim Morschhausser) [40] with primers TCO42+TCO43, with BamHI at both ends and it was cloned into pTC19. The resultant pTC20 was then linearized with *XcmI*, and transformed into wild-type SC5314.

To generate the heterozygous deletion of *PKC1*, ~500 bp 5’ and 3’ untranslated regions (UTRs) flanking *PKC1* were amplified as *KpnI-Xho*I and *NotI-SacI* fragments, by using the primers TCO59+TCO60 and TCO62+TCO110, respectively. The 5’ and 3’ UTR fragments were cloned into the corresponding sites of the SAT1 flipper in the plasmid pSFS2A (Reuss, et al, 2004), respectively. The resulting plasmid pTC041 was digested with *KpnI* and *SacI*, and the larger fragment was gel purified (Qiagen Inc.) and transformed into the strains of interest. The nourseothricin-resistant transformants were further cultured in medium containing maltose as a carbon source to induce *caFLP* expression, which is under *MAL2* promoter regulation. The confirmation of a *PKC1* allele being deleted was verified with TCO63 + JCO95.

To introduce the Q67L mutation into *RHO1,* first the *RHO1* gene was PCR amplified with primers TCO8+TCO72 from SC5314 genomic DNA as a *SacI-NotI* fragment and cloned into vector pBT1 (Tams, et al, In Press) to generate pTC35. Then primers TCO74+TCO75 were used to create pTC38 from pTC35 by point mutating *RHO1* using site-directed mutagenesis. A similar site directed mutagenesis protocol was used to introduce the G12V mutation into *CDC42.* TRO996+TCO101 were used for *CDC42* PCR amplification and it was cloned into pBT1 to create pTC34. Then TCO76+TCO77 were used to introduce the G12V mutation to *CDC42* to result in pTC37 which expresses *P_ENO1_*- *CDC42^G12V^.* pTC37 and pTC38 were linearized with *MscI* and transformed into SC5314.

To create the strain that has GFPCRIB (Cdc42-Rac1 interactive binding motif) to probe the localization of GTP-Cdc42, *RAC1* was disrupted in SC5314 using *C. albicans* CRISPR-Cas9. This was to exclude GFPCRIB binding with Rac1. Primers TCO38+TCO39 were annealed to generate a *RAC1* sgRNA flanked with BsmbI, which were then ligated with the CRISPR deletion construct pV1393 [45] to create the plasmid pTC18. TCO40+TCO41 were used to generate the repair template that spanned the Cas9 cut site in order to introduce 3 stop codons to prevent *RAC1* expression and a new *HindIII* cut site to allow quick genotyping of the transformants. pTC18 was digested with *KpnI* and *SacI*, and the larger fragment was transformed into both SC5314 and *cho1∆/∆* together with repair template. TCO51+TCO52 were used for colony PCR to test if the repair template had integrated into the Cas9 cut site. The positive *rac1ΔΔ* transformant was then transformed with linearized pTC029. The pTC029 plasmid was created from pExpArg-pADH1CRIBGFP [44] by cutting it with NotI and ligating it with a *Candida*-adapted hygromycin B resistance marker amplified with primers TCO92/TCO93 from pRB436 (a gift from Dr. Richard Bennett). CRISPR/Cas9 was also utilized to knock out *MKC1* and *PKC1* in the strains of interests. TCO32+TCO33 and TCO44+TCO45 were used to generate the sgRNA for *MKC1* and *PKC1*, respectively. TCO34+TCO35, TCO46+TCO47 were used to create the repair templates for *MKC1* and *PKC1* with *EcoRI* and *BglII* introduced, respectively. TCO28+TCO29 and TCO49+TCO50 were used for colony PCR on transformants, respectively to check if the repair template integrated into the Cas9-cut region.

To generate strains that have GFPRID (Rho1 Interactive Domain) to probe the localization of GTP-Rho1, each strain was transformed with pTC033. The pTC033 plasmid consists of pExpArg-pACT1GFPRID [44] with a SAT1 marker cloned into the Not I restriction site following amplification with TCO15/TCO16. The pTC033 plasmid was linearized with StuI and transformed into the strains of interest.

**Supplemental References**

De Backer MD, Maes D, Vandoninck S, Logghe M, Contreras R, Luyten WH. Transformation of Candida albicans by electroporation. Yeast. 1999;15(15):1609-18. doi: 10.1002/(SICI)1097-0061(199911)15:15<1609::AID-YEA485>3.0.CO;2-Y. PubMed PMID: 10572258.

Gillum AM, Tsay EY, Kirsch DR. Isolation of the Candida albicans gene for orotidine-5'-phosphate decarboxylase by complementation of S. cerevisiae ura3 and E. coli pyrF mutations. Mol Gen Genet. 1984;198(2):179-82. PubMed PMID: 6394964.

Reuss O, Vik A, Kolter R, Morschhauser J. The SAT1 flipper, an optimized tool for gene disruption in Candida albicans. Gene. 2004;341:119-27. doi: 10.1016/j.gene.2004.06.021. PubMed PMID: 15474295.

Tams RT, Cassilly CD, Anaokar S, Brewer WT, Dinsmore J, Chen YL, Patton-Vogt J, Reynolds TB. Overproduction of Phospholipids by the Kennedy Pathway Leads to Hypervirulence in Candida albicans. Frontiers in Microbiology. In Press.
