# Supplementary figures and images for: Exposure of Candida albicans β (1,3)-glucan is promoted by activation of the Cek1 pathway
Source: PLoS Genet. 2019 Jan 31;15(1):e1007892. doi: 10.1371/journal.pgen.1007892 (PMC6372213; doi:10.1371/journal.pgen.1007892)

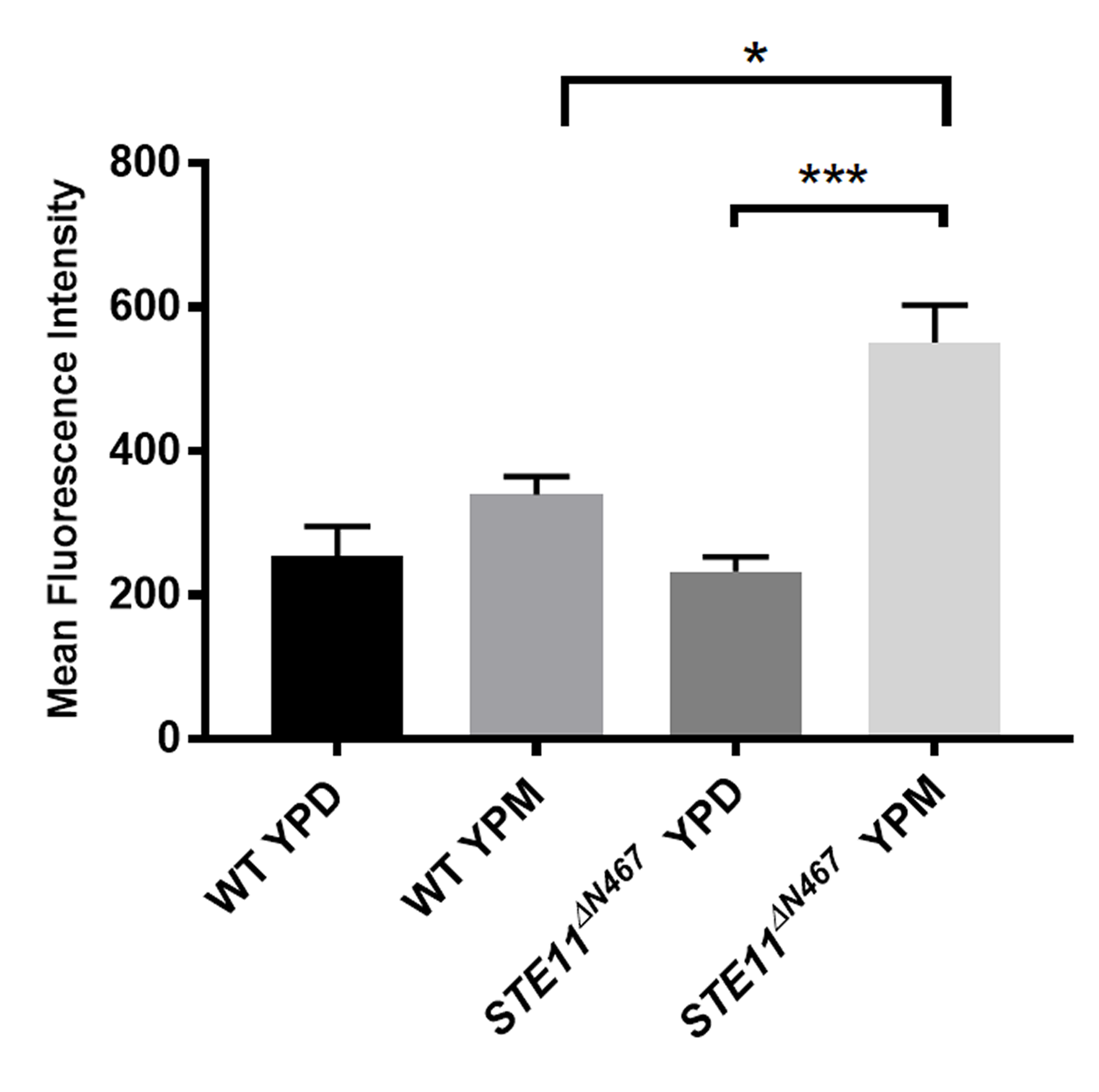

Supplement: S1 Fig — Overnight cultures of Candida cells was incubated with anti-β (1,3)-glucan primary antibody and PE-conjugated secondary antibody, followed by flow cytometry to quantify the fluorescence intensity. Data represent three biological replicates. The statistical analysis was done by One-way ANOVA. ***, P = 0.0004; *, p = 0.0137 (TIF) [file pgen.1007892.s005.tif]

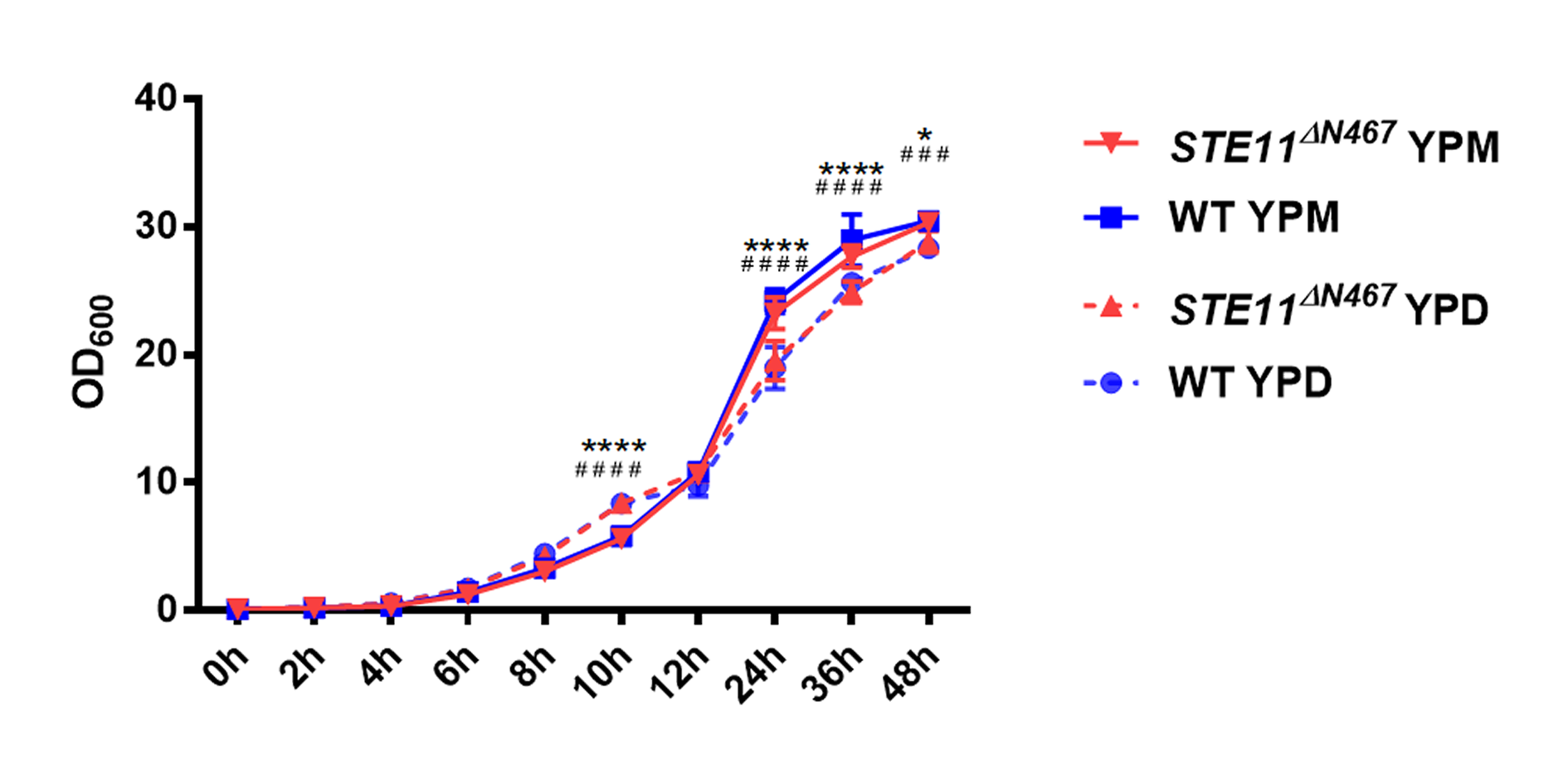

Supplement: S2 Fig — Cells were grown overnight in YPD, diluted back to 0.1 OD600 and transferred to fresh YPD or YPM. A growth curve was performed with three replicates per condition, and plotted based on the growth rate of different strains measured in 48 hrs. The growth at each time-point between YPD and YPM cultures of STE11ΔN467 were compared by Two-way ANOVA(****, p<0.0001; *, p = 0.0286). The same comparison was made between wild-type YPD and YPM culture (####, p<0.0001; ###, p = 0.0007). (TIF) [file pgen.1007892.s006.tif]

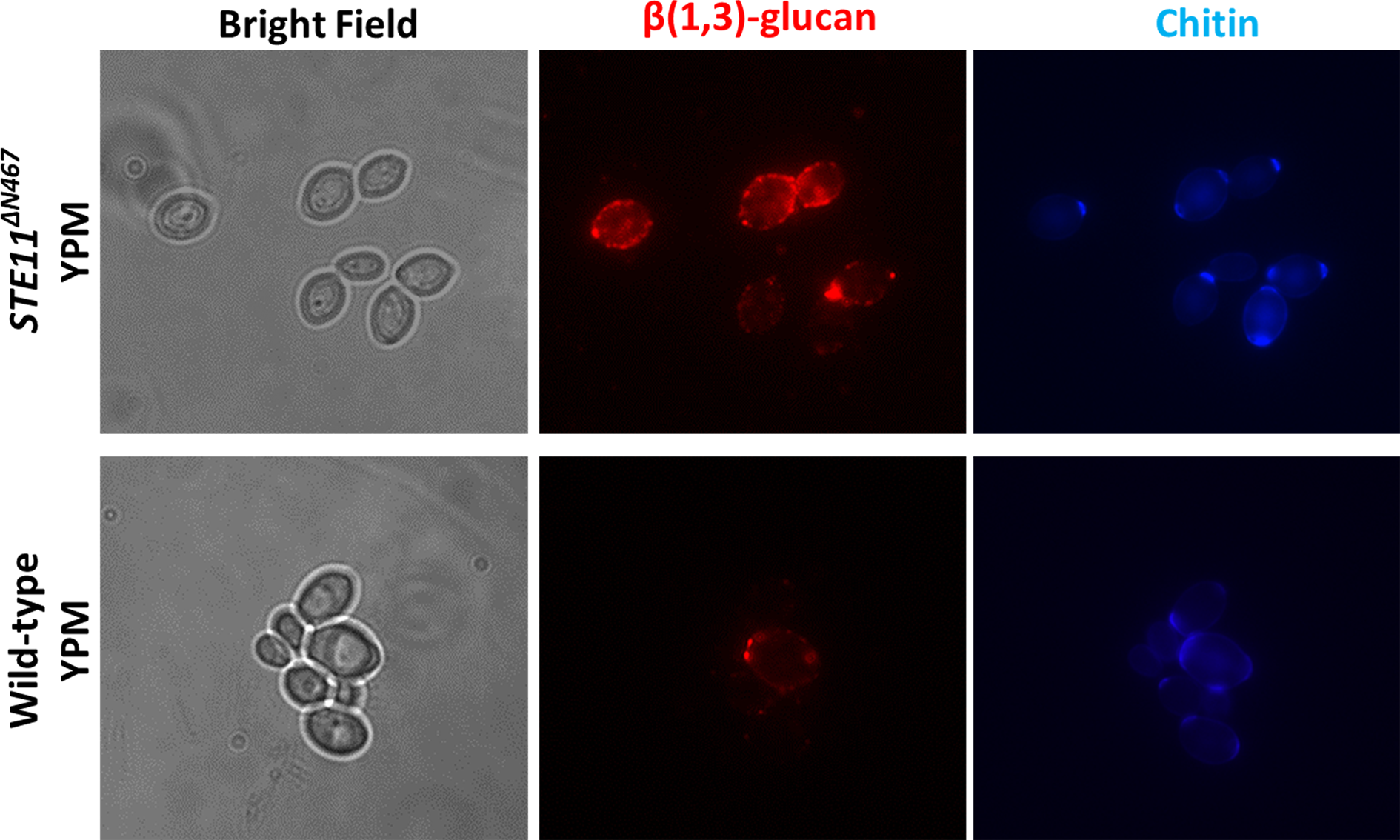

Supplement: S3 Fig — Overnight cultures of wild-type and STE11ΔN467 grown in YPM were co-stained with anti-β(1,3)-glucan antibody and Cy3 secondary to visualize exposed β(1,3)-glucan and calcofluor white to visualize chitin. (TIF) [file pgen.1007892.s007.tif]

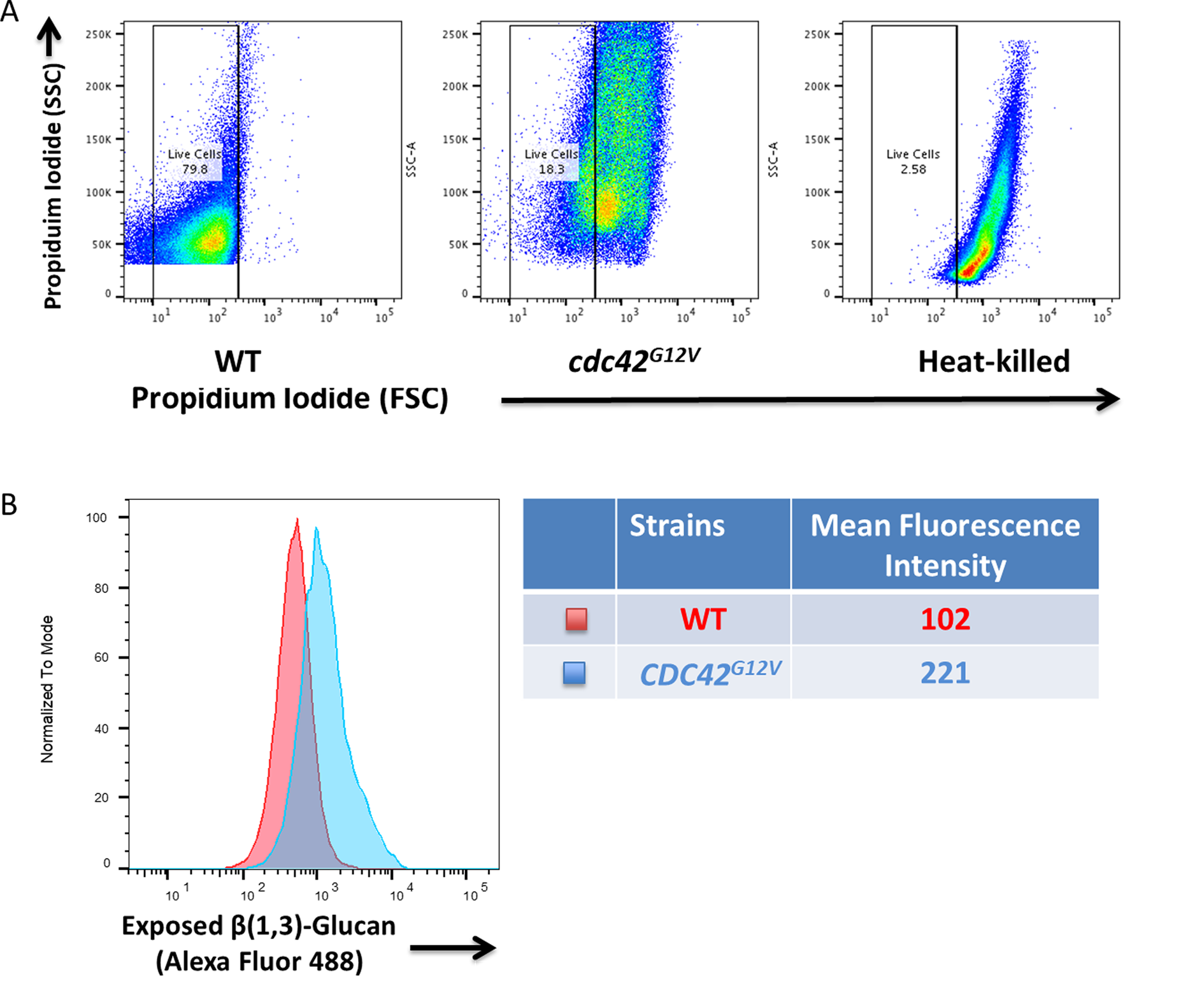

Supplement: S4 Fig — (A) Propidium iodide staining was performed to quantify the live cells in Candida strains. (B) β (1,3)-glucan exposure in live (gated for propidim iodide negative cells) wild-type and CDC42G12V populations was measured by flow cytometry. (TIF) [file pgen.1007892.s008.tif]

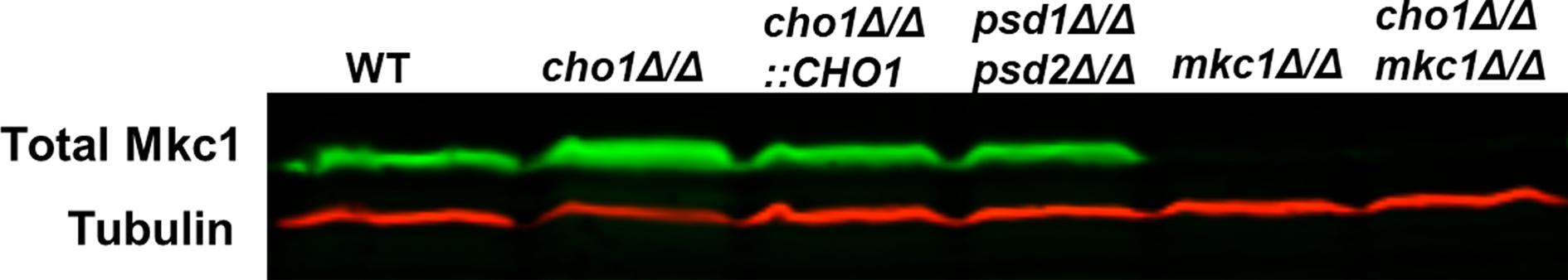

Supplement: S5 Fig — Western blotting was performed using anti-Mkc1 antibody to confirm the absence of Mkc1 in the MKC1 knockout mutants compared to wild-type (WT) and other strains. Tubulin was probed with anti-tubulin antibody as a loading control. (TIF) [file pgen.1007892.s009.tif]

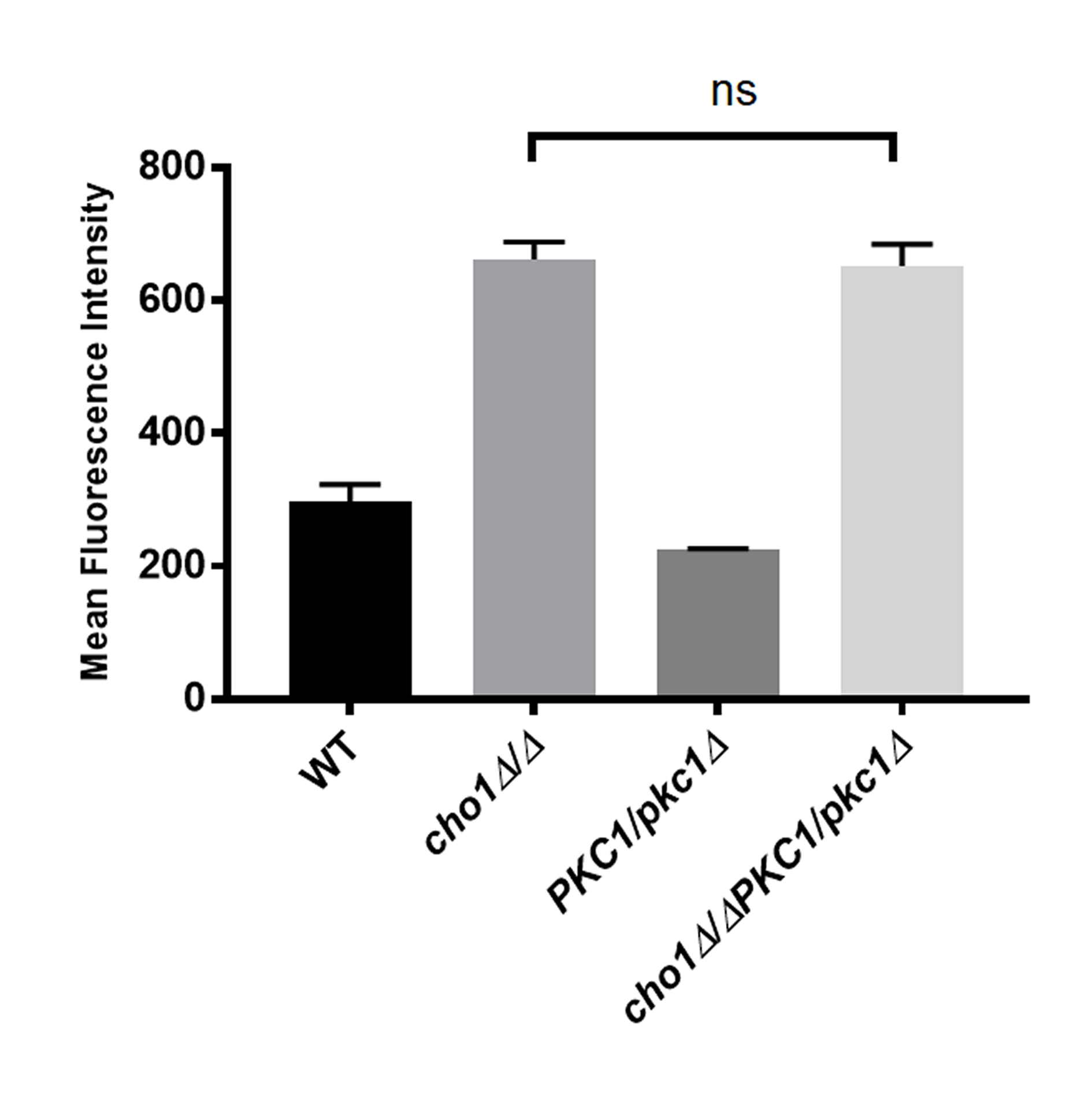

Supplement: S6 Fig — One PKC1 allele was deleted by the SAT1-flipper method. Cells were then stained with anti-β (1,3)-glucan primary antibody and phycoerythrin (PE)-conjugated secondary antibody. The statistical analysis was carried out by doing One-way ANOVA. (TIF) [file pgen.1007892.s010.tif]
